# *In vitro* Modeling of Chicken Cecal Microbiota Ecology and Metabolism Using the PolyFermS Platform

**DOI:** 10.3389/fmicb.2021.780092

**Published:** 2021-12-20

**Authors:** Paul Tetteh Asare, Anna Greppi, Alessia Pennacchia, Katharina Brenig, Annelies Geirnaert, Clarissa Schwab, Roger Stephan, Christophe Lacroix

**Affiliations:** ^1^Laboratory of Food Biotechnology, Department of Health Sciences and Technology, Institute of Food, Nutrition and Health, ETH Zürich, Zurich, Switzerland; ^2^Institute for Food Hygiene and Safety, University of Zurich, Zurich, Switzerland

**Keywords:** *in vitro* model, broiler, cecum, microbiota, PolyFermS

## Abstract

Continuous *in vitro* fermentation models provide a useful tool for a fast, reproducible, and direct assessment of treatment-related changes in microbiota metabolism and composition independent of the host. In this study, we used the PolyFermS model to mimic the conditions of the chicken cecum and evaluated three nutritive media for *in vitro* modeling of the chicken cecal microbiota ecology and metabolism. We observed that our model inoculated with immobilized cecal microbiota and fed with a modified Viande Levure medium (mVL-3) reached a high bacterial cell density of up to approximately 10.5 log cells per mL and stable microbiota composition, akin to the host, during 82 days of continuous operation. Relevant bacterial functional groups containing primary fibrolytic (*Bacteroides*, *Bifidobacteriaceae*, *Ruminococcaceae*), glycolytic (*Enterococcus*), mucolytic (*Bacteroides*), proteolytic (*Bacteroides*), and secondary acetate-utilizing butyrate-producing and propionate-producing (*Lachnospiraceae*) taxa were preserved *in vitro.* Besides, conserved metabolic and functional Kyoto Encyclopedia of Genes and Genomes pathways were observed between *in vitro* microbiota and cecal inoculum microbiota as predicted by functional metagenomics analysis. Furthermore, we demonstrated that the continuous inoculation provided by the inoculum reactor generated reproducible metabolic profiles in second-stage reactors comparable to the chicken cecum, allowing for the simultaneous investigation and direct comparison of different treatments with a control. In conclusion, we showed that PolyFermS is a suitable model for mimicking chicken cecal microbiota fermentation allowing ethical and *ex vivo* screening of environmental factors, such as dietary additives, on chicken cecal fermentation. We report here for the first time a fermentation medium (mVL-3) that closely mimics the substrate conditions in the chicken cecum and supports the growth and metabolic activity of the cecal bacterial akin to the host. Our PolyFermS chicken cecum model is a useful tool to study microbiota functionality and structure *ex vivo*.

## Introduction

The microbial community in the chicken (i.e., broiler and hen) gastrointestinal tract (GIT) plays an essential role in shaping health and productive performance (Oakley et al., 2014; Stanley et al., 2014). The most densely populated section within the chicken GIT are the ceca, a pair of blind-ended sacs that open off the large intestine (Clench and Mathias, 1995; Rychlik, 2020). The cecum, because of its more extensive and diverse microbial population and longer transit time of its digesta (12–24 h), is the main region for bacterial fermentation but also the main site for the colonization of pathogens (Sergeant et al., 2014; Stanley et al., 2015). Therefore, the majority of chicken microbiota studies focused primarily on the cecal microbial communities (Glendinning et al., 2020). The chicken cecal microbiota is dominated by the phyla Firmicutes, Bacteroidetes, and Proteobacteria (Wei et al., 2013; Rychlik, 2020) and has been implicated in nitrogen recycling by the breakdown of uric acid (Karasawa, 1999) and the supply of B vitamins and essential amino acids to the host (Sergeant et al., 2014). In addition, short-chain fatty acids (SCFAs), mainly acetate, propionate, and butyrate, are produced by microbial fermentation of undigested carbohydrates reaching the cecum (Józefiak et al., 2004). SCFAs contribute to the energy supply, improve mineral absorption, inhibit the growth of acid-sensitive pathogens, and have systemic health effects upon epithelial absorption (Kumar et al., 2019).

*In vivo* chicken trials have been adopted and used to study the role of the animal’s microbiota in nutrient utilization, health, performance, and product (i.e., meat and eggs) quality (Oakley et al., 2014; Celi et al., 2017; Borda-Molina et al., 2018; Yadav and Jha, 2019). However, mechanistic studies in chicken can be confounded as it is not possible to focus solely on the cecal microbiota because of the presence of the host and host-related factors (Kers et al., 2018; Wen et al., 2021). The presence of the host makes mechanistic studies more complicated, as it might not be clear whether an intervention directly alters the structure or functionality of the gut microbiota, or if it elicits a response by the host, which then indirectly alters the gut microbiota (Van Den Abbeele et al., 2010; McDonald, 2017). Besides, chicken trials are expensive and difficult to control, and data are often derived from end-point measurements, requiring animal sacrifice at each time point. For ethical reasons, there is a need to reduce animal experiments, and the administration of untested or unsafe compounds is prohibited (Macfarlane and Macfarlane, 2007; Akhtar, 2015). Therefore, *in vitro* microbiota models are adopted to help elucidate bacterial processes occurring within the cecal microbiome and to overcome some of the limitations of *in vivo* models.

Intestinal *in vitro* modeling systems are suitable for the study of non–host-associated factors that shape bacterial interactions and metabolism in a specific niche, without ethical constraints (Lacroix et al., 2015). *In vitro* gut fermentation models aim to cultivate a complex intestinal microbiota under controlled conditions, akin to the host, and to carry out microbial modulation and metabolism studies. They are important components of multiscale strategies to investigate mechanisms or functions of dietary compounds on the gut microbiota, host health, and physiology (Payne et al., 2012; Lacroix et al., 2015). The successful transfer, adaptation, survival, and proliferation of *in vivo*–acquired gut microbiota to *in vitro* fermentation systems depend on the strict control of environmental parameters (temperature, pH, retention time, anaerobiosis) of the source host (Payne et al., 2012; Verhoeckx et al., 2015). Proper control of these environmental factors facilitates the establishment of steady-state conditions in terms of both microbial composition and metabolic activity in *in vitro* systems (Payne et al., 2012; Venema and Van Den Abbeele, 2013).

A range of systems have been developed to model fermentation of the GIT, from simple anaerobic batch culture systems in flasks to multistage continuous flow models, (Pham and Mohajeri, 2018; Fournier et al., 2021). Batch culture systems are limited to short-term fermentation experiments. The continuous-flow PolyFermS model allows for the stable cultivation of complex intestinal microbiota over extended periods of several months and for the comparison of experimental factors in second-stage parallel reactors inoculated with the same microbiota composition produced in the first-stage reactor inoculated with immobilized microbiota (Zihler Berner et al., 2013; Fehlbaum et al., 2015). The model was developed to prevent the rapid washout of less dominant and slow-growing bacteria species and cultivates both the sessile and planktonic states of the microbiota over a long period of up to 150 days (Payne et al., 2012; Pham et al., 2019; Isenring et al., 2021). PolyFermS models mimicking different hosts and conditions were developed and used for ecological and mechanistic studies of infant, child, adult, and elderly human microbiota and swine and murine gut microbiota (Zihler Berner et al., 2013; Tanner et al., 2014a; Dostal et al., 2015; Doo et al., 2017; Pham et al., 2019; Poeker et al., 2019).

Until now, *in vitro* chicken cecal continuous fermentation models have been little investigated and applied, whereas most research was done *in vivo* (Mota de Carvalho et al., 2021; Oost et al., 2021). Card et al. (2017) and Gong et al. (2019) have described and implemented a continuous culture of the chicken ceca microbiota inoculated with free-cell suspension in single-stage chemostat fermentation. The challenge with the use of free-cell suspension is the loss of less dominant, slow-growing, or sessile bacteria species by rapid washout and the low cell density measured in the fermentation vessel compared with *in vivo.* Low cell density can highly impact competition and cross-feeding among bacteria, leading to a significant difference in both the α and β diversity over time (Card et al., 2017). Very recently, Oost et al. (2021) reported a new *in vitro* cecal chicken dynamic model developed on the TIM 2 platform. The model used a pooled frozen inoculum that was cryopreserved with addition of 15% glycerol. A significant shift in bacterial composition was reported compared with the inoculum. Furthermore, in the short-term 72-h operation of the model, no differences in the microbial composition and metabolite production were detected when testing different fermentation medium composition mimicking the ileum chyme, including the sources of starch, fibers, and simple sugars. This stresses the need to develop improved models for chicken cecal fermentation.

It was therefore the aim of this study, to develop a continuous flow *in vitro* fermentation model inoculated with immobilized chicken cecal microbiota based on the PolyFermS platform that closely mimics the composition and activity of chicken cecal microbiota and can be applied to evaluate dietary and environmental factors. We first analyzed the microbiota composition and metabolic profile in the cecum of chicken to select the conditions applied in the model based on *in vivo* data. We improved stepwise the formulation of the standard Viande Levure medium (mVL) that was previously used for *in vitro* fermentation of chicken gut microbiota, to mimic the substrate conditions in the chicken cecum and support the growth and metabolic activity of the cecal bacterial akin to the host cecum. Finally, we evaluated the long-term stability and the reproducibility of the microbiota and activity within the reactors of a set model.

## Materials and Methods

### Cecal Collection

For establishing *in vivo* data of the microbiota composition and metabolic profile in the cecum of chicken, 10 complete GITs of freshly slaughtered Cobb-500 broiler chicken (35 days old) were obtained from the slaughter plant of Bell Food Group (Zell, Switzerland) under the supervision of a veterinary officer. Upon collection, the samples were kept chilled (<8°C) and immediately transported to the laboratory for analysis. The ceca were removed from the whole GIT and placed on a sterile Petri dish. The cecal content was collected into DNAse-free tubes, and the pH was immediately measured using a probe precalibrated pH meter (Metrohm 744 pH meter; Metrohm AG, Herisau, Switzerland). The cecal content was afterward snap-frozen in liquid nitrogen and stored at −20°C until DNA isolation and metabolite extraction.

For the model inoculation, fresh ceca from healthy 21-day-old Cobb-500 broiler chicken were collected under the supervision of a veterinary officer from three different local chicken farms in Zurich, Switzerland. The broiler chicken was not treated with antibiotics before and not feed restricted. The chicken was stunned; the entire GIT was removed, placed in sterile container with an Oxoid AnaeroGen strip (Oxoid, England, United Kingdom), and transported on ice to the laboratory within 1 h after collection of the GIT. In the laboratory, the entire GIT was transferred into an anaerobic chamber (Coy Laboratories, Ann Arbor, MI, United States) for immobilization.

### *In vitro* Fermentation Model

#### Nutritional Medium

A nutritive medium was formulated, with modifications from the standard VL medium that was previously used for *in vitro* fermentation of chicken gut microbiota (Yin et al., 2010; Lei et al., 2012; Card et al., 2017; Gong et al., 2019). The VL medium contains mainly hydrolyzed nitrogen sources and simple sugars with a protein-to-carbohydrate ratio of 7:1. This medium lacks, however, minerals, trace elements, and soluble carbohydrates expected in the ceca, which may support the growth and metabolism of the chicken cecal microbiota (Svihus et al., 2013; Svihus, 2014). Therefore, the VL medium was supplemented with Tween 80, bile salts, minerals, and vitamins based on the validated human intestinal microbiota growth medium described by Macfarlane et al. (1998). Porcine mucin (2.0 g/L) was also added to mimic the contribution of endogenous mucin secretion. The composition of the mVL-1 medium is presented in Supplementary Table 1.

The mVL-1 medium was further supplemented with 2.5 g/L of fructooligosaccharides (FOS) (mVL-2) that have been shown to support the growth of *Bifidobacterium* and *Lactobacillus* species groups when added to the chicken diet (Liu et al., 2017; Kumar et al., 2019). Based on the results obtained, the mVL-2 medium was further supplemented with pectin from citrus (2.5 g/L) to promote the colonization and proliferation of Firmicutes and Bacteroidetes (mVL-3 medium). Pectins are plant cell-wall polysaccharides that can be utilized by commensal bacteria in the gut belonging to the phyla Firmicutes and Bacteroidetes (Larsen et al., 2019).

The media base constituents and FOS (Fibrulose F97; Cosucra Group, Warcoing, Belgium) were dissolved in distilled water, adjusted with 5 M NaOH to pH 6, autoclaved at 121°C for 20 min, and stored at 4°C, with stirring until needed. After cooling to 4°C, 1 mL of a filter-sterilized (0.2-μm pore size) vitamin solution (Michel et al., 1998) was added to the medium. For the initial bead colonization carried out in batch culture, the media were supplemented with a mixture of SCFAs, with final concentrations of acetate, propionate, and butyrate of 31, 9, and 10 μmol/mL, respectively, based on concentration measured for the *in vivo* cecal metabolite, to prevent the overgrowth of fast-growing bacteria.

All components were purchased from Sigma–Aldrich Chemie (Buchs, Switzerland), except bile salts (Oxoid AG), yeast extract (Merck, Darmstadt, Germany), NaHCO_3_ (Fischer Scientific, Pittsburgh, PA, United States), NaCl and KH_2_PO_4_ (VWR International AG, Dietikon, Switzerland), MgSO_4_⋅anhydrous (Acros Organics, Geel, Belgium), and MnCl_2_⋅4H_2_O (Fluka, Buchs, Switzerland).

#### Cecal Microbiota Immobilization and Bead Colonization

For each immobilization procedure, the cecal content was obtained from 21-day-old Cobb-500 broiler chicken as presented previously. We used the 21-day chicken cecum microbiota to prime the model and operate under the set conditions of the cecum because this age corresponded to a stable microbiota in broiler chicken and would allow for testing different nutritional factors during the growth phase (Mohd Shaufi et al., 2015; Ijaz et al., 2018; Jurburg et al., 2019).

The ceca were removed from the GIT, and the cecal content was squeezed into an empty sterile tube and mixed with 0.1 M anaerobic phosphate-buffered saline (pH 7.2) to create a 10% (w/v) slurry in anaerobic conditions (10% CO_2_, 5% H_2_, and 85% N_2_) (Anaerobic Chamber; Coy Laboratories). The cecal microbiota was immobilized in gel beads using a double-phase dispersion process as described before (Zihler Berner et al., 2013; Pham et al., 2019).

The entire immobilization procedure was carried out under anaerobic conditions. Briefly, 10 mL cecal slurry was added to 500 mL of sterile polymer mix consisting of gellan gum (2.5%, w/v), xanthan (0.25%, w/v), and sodium citrate (0.2%, w/v). The inoculated polymer solution was added to sterile sunflower oil at 40°C under agitation with a magnetic stirrer set to target bead diameters in the 1- to 2-mm range, and the resulting macroemulsion was cooled down to 30°C to induce gel formation. The gel beads were washed and soaked in a sterile solution containing 100 mM CaCl_2_ (Sigma–Aldrich). Beads with a diameter of 1–2 mm were selected by wet sieving under sterile conditions.

The immobilized chicken cecal microbiota beads (60 mL) were immediately transferred under anaerobiosis to a glass bioreactor (Multifors; Infors AG) containing 140 mL of sterile and anaerobic fermentation medium supplemented with SCFA mixture (Table 1A). To ensure initial bead colonization, two consecutive batch fermentations of 20 and 6 h were carried out in fully controlled bioreactors operated at a volume of 200 mL with stirring set at 180 revolutions/min. After the first batch incubation, 100 mL of the spent medium was exchanged with the same volume of fresh medium. Anaerobiosis was generated by continuous flushing of the headspace of the reactor and fermentation medium with pure filter sterile CO_2_. The temperature was controlled at chicken body temperature of 41°C, and the pH was maintained at 6.0 by automatic addition of 2.5 M NaOH.

**TABLE 1:** Conditions of initial bead batch colonization (A) and continuous fermentation (B) for different continuous cecal microbiota fermentations.

(A) Batch fermentation conditions for initial bead colonization				
	F1-A	F1-B	F2	F3
Nutritive medium*	mVL-1	mVL-2	mVL-3	mVL-3
Supplementation	SCFA mix	SCFA mix	SCFA mix	SCFA mix
pH	6	6	6	6
Colonization—batch 1	20 h	20 h	20 h	20 h
Colonization—batch 2	6 h	6 h	6 h	6 h

**(B) Continuous fermentation conditions**				

	**F1-A**	**F1-B**	**F2**	**F3**

Nutritive medium*	mVL-1	mVL-2	mVL-3	mVL-3
pH	6	6	6	6
Retention time	24 h	24 h	24 h	24 h
Total fermentation time (days)	13	13	70	82

*Composition presented in Supplementary Table 1. mVL-1, modified Viande Levure medium. mVL-2, modified Viande Levure medium with 2.5 g/L FOS. mVL-3, modified Viande Levure medium with 2.5 g/L FOS and 2.5 g/L citrus pectin.

#### Continuous Cecal Microbiota Fermentation

After the initial colonization of cecal beads, the reactors were switched to continuous fermentation mode by the continuous supply of fresh, sterile, and anaerobic nutritive medium and the removal of the equivalent volume of fermented medium with peristaltic pumps (Reglo; Ismatec, Glattbrugg, Switzerland) and operated under the same conditions as for the batch cultures (Table 1A). The continuous flow rate of the fresh and spent medium was set at 8.4 mL/h corresponding to a mean retention time (RT) of 24 h selected to mimic the infrequent emptying of the ceca (Hinton et al., 2000; Warriss et al., 2004; Yin et al., 2010).

#### Experimental Design

**FIGURE 1:**
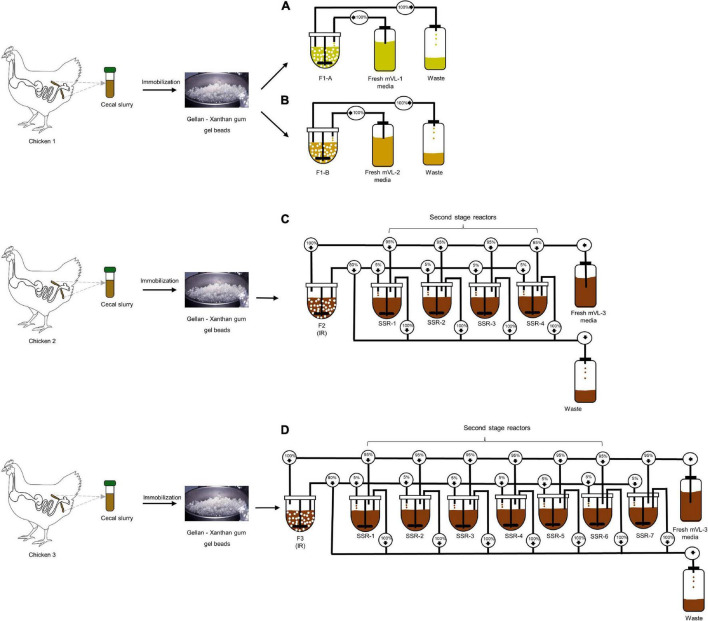
Experimental design of *in vitro* continuous PolyFermS fermentations inoculated with immobilized chicken cecal microbiota. Single cecal microbiota was immobilized in a gellan–xanthan gel for each fermentation. **(A,B)** The immobilized cecal microbiota of chicken 1 was used to inoculate two bioreactors (30% v/v); F1-A and F1-B and continuously fed with mVL-1 and mVL-2 fermentation medium, respectively. **(C)** F2 consisted of an inoculum reactor (IR) containing cecal beads of chicken 2, connected to four second-stage reactors (SSR) and continuously fed with 5% fermentation effluent of IR and 95% fresh fermentation mVL-3 medium. **(D)** F3 consisted of an inoculum reactor (IR) containing cecal beads of chicken 3, connected to seven second-stage reactors (SSR) and continuously fed with 5% fermentation effluent of IR and 95% fresh fermentation mVL-3 medium. For activity, microbial composition and metabolites in reactor effluent samples were monitored daily using MiSeq and HPLC-RI, respectively, to show to the model stability and compared with the cecal inoculum.

Four cecal continuous fermentation experiments were performed as illustrated in Figure 1. The inoculum reactor (IR) of F1-A and F1-B were inoculated with the same cecal beads and operated in continuous mode for 13 days with mVL-1 and mVL-2 medium, respectively (Figures 1A,B).

For F2, the IR was operated in continuous mode for 70 days with mVL-3 medium to evaluate the long-term stability of the *in vitro* model. Stability was defined as the period with less than 10% variations in the daily metabolite concentrations. After an initial stabilization of 20 days, IR of F2 was connected to four second-stage reactors (SSRs), continuously inoculated with 5% (v/v) fermentation effluent produced in IR, and additionally supplied with 95% fresh mVL-3 medium (period 1). This design of PolyFermS allows to generate similar microbiota composition and activity in the SSRs inoculated with the same microbiota generated in IR and is useful for testing different biotic and abiotic factors on the modeled microbiota (Tanner et al., 2014a; Fehlbaum et al., 2015). The reactors were operated for 10 days with the same conditions as IR (Figure 1C). F3, composed of IR and seven SSRs, was a repetition of F2, and carried out for 82 days with a new cecum inoculum (Figure 1D).

Effluent samples were taken daily from each reactor and separated into bacterial pellet (10 min at 14,000 × *g* at 4°C) and supernatant and stored at −20°C until further analysis. The stability of the fermentation was monitored by daily measurements of the main fermentation metabolite concentrations in effluent sample supernatants.

### High-Performance Liquid Chromatography With Refractive Index Detector Analysis of Microbial Metabolites

SCFA (acetate, propionate, butyrate, formate, valerate), branched-chain fatty acids (BCFAs; isobutyrate and isovalerate), and intermediate metabolites (succinate and lactate) concentrations in the cecal and fermentation effluent samples were determined by high-performance liquid chromatography with refractive index detector (HPLC-RI) analysis. Analyses were performed with an Accela Chromatography System and RI detector (Thermo Fisher Scientific Inc., Reinach, Switzerland), equipped with a Security Guard Carbo-H cartridge (4 mm × 3.0 mm) and a Rezex ROA-Organic Acid H + column (300 mm × 7.8 mm). The column was eluted with 10 mM H_2_SO_4_ (Fluka) as a mobile phase at a flow rate of 0.4 mL min^–1^ at 25°C. Cecal samples (500 mg) were mixed with 1 mL of 0.5 mM H_2_SO_4_, homogenized, and centrifuged at 4°C at 9,000 × *g* for 20 min. The supernatant was filtered using 0.45-μm nylon membrane (Millipore AG, Zug, Switzerland) into glass HPLC vials (Infochroma AG, Zug, Switzerland). Reactor effluent supernatant samples were filtered into glass HPLC vials (Infochroma) through a 0.45-μm nylon membrane (Millipore AG) and sealed with crimp caps. SCFAs, BCFAs, and intermediate metabolite concentrations were quantified using external standards (Sigma–Aldrich Chemie).

### Microbial Community Analysis

#### Genomic DNA Extraction

Total genomic DNA was extracted from 500 mg cecal sample and pellet of 2 mL fermentation effluent using the FastDNA^®^ SPIN Kit for Soil (MP Biomedicals, Illkirch Cedex, France) and final elution volume of 100 μL according to the manufacturer’s instructions. DNA concentrations were determined using a Nanodrop^®^ ND-1000 Spectrophotometer (Wiltec AG, Littau, Switzerland), and samples were stored at −20°C until analysis.

#### Quantitative Polymerase Chain Reaction Analysis

Polymerase chain reaction (PCR) analysis of total bacteria and selected bacterial groups commonly found in broiler cecum was performed with specific primers (Supplementary Table 2), synthesized by Microsynth AG (Balgach, Switzerland), targeting Firmicutes, Bacteroidetes, *Ruminococcaceae*, *Lactobacillus–Leuconostoc–Pediococcus*, *Bifidobacteriaceae*, and *Enterobacteriaceae* species. The diluted DNA (1 μL) was used for amplification in duplicate in 20 μL reaction solution, containing 10 μL of SensiFAST SYBR No-ROX Kit (Bioline, Luckenwalde, Germany) and 10 pmol of each primer. Quantitative PCR (qPCR) reactions were performed using a Roche LightCycler 480 II (Roche Diagnostics AG, Rotkreuz, Switzerland). Reactions were preincubated in LightCycler 480 Multiwell plate 96 (Roche Diagnostics AG) at 95°C for 3 min, followed by 45 cycles at 95°C for 5 s and 60°C for 30 s. At the end of the qPCR cycles, melting curve analysis was performed to validate the specific generation of the expected PCR products. Each reaction was run in duplicate. For quantification, dilution series of standards obtained by amplification of the linearized plasmid containing the representative gene of the target bacterial species were included in each run. qPCR data were analyzed using the LightCycler^®^ 480 Software 1.5.1 (Roche Diagnostics AG). PCR efficiency (%) was calculated from the slope of the standard curve for each qPCR assay. Assays with an efficiency of 80% to 110% (slope 3.2–3.9) were retained.

#### Microbiota Profiling With 16S rRNA Amplicon Sequencing

Bacterial communities in cecal and fermentation effluent samples were determined using the Illumina MiSeq platform (Genetic Diversity Center, ETH Zurich) using an in-house protocol. In F1-A, F1-B and F2 effluent samples were amplified and sequenced using universal primers targeting the V3 region. F3 samples and inoculum cecal microbiota were analyzed with primers targeting the V4 region of the 16S rRNA gene. The sequencing of samples obtained from F3 was performed primarily to evaluate the microbial ecology *in vitro* to the cecal inoculum but not with previous fermentations (F1 and F2) because different 16S rRNA gene regions were used. Primers 388F (5′-ACWCCTACGGGWGGCAGCAG-3′) and 518R (5′-ATTACCGCGGCTGCTGG-3′) were used to amplify the V3 region (Muyzer et al., 1993). Primers 515F (5′-GTGCCAGCMGCCGCGGTAA-3′) and 806R (5′-GGACTACHVGGGTWTCTAAT-3′) were used for the V4 region (Caporaso et al., 2011). PCR reactions were carried out with 10 μL 2X KAPA HiFi HostStart ReadyMix (Roche Diagnostics AG), 0.5 μL of each primer (10 μM), 2 μL of genomic DNA (∼20 ng/μL), and 7 μL nuclease-free water to a total volume of 20 μL, and run on a SensoQuest LabCycler Basic 96 (Witec AG, Sursee, Switzerland). Cycling conditions applied were as follows: denaturation at 95°C for 3 min, 25 cycles of 98°C for 20 s (denaturation), 55°C for 15 s (annealing), 72°C for 15 s (extension), and final extension of 72°C for 2 min. To incorporate primers with adapters, PCR reactions contained 10 μL 2X KAPA HiFi HostStart ReadyMix (Roche Diagnostics AG), 2 μL corresponding adapter primers (Nextera Index Kit), 2 μL PCR product, and nuclease-free water to a total volume of 25 μL. Cycling conditions applied were as follows: denaturation at 95°C for 3 min, 12 cycles of 95°C for 30 s, 55°C for 30 s, and 72°C for 30 s, followed by final elongation of 72°C for 5 min. The amplified fragment with adapters was purified using AMPure XP beads (Beckman Coulter Genomics, Brea, CA, United States). The prepared library was quantified using Qubit fluorometer (Invitrogen, Carlsbad, CA, United States), and approximately equal concentrations were pooled together using Liquid Handling Station (Brand, Hamburg, Germany).

The pooled libraries were denatured into single-stranded molecules with freshly made 0.2 N NaOH and diluted at 12 pM before being mixed with 20% of Illumina PhiX control libraries. The mixed Phix/16S libraries were sequenced in multiplex on the MiSeq machine with a V2 reagent kit for 2 × 250 bp paired-end Nextera chemistry. Sequence demultiplexing was performed automatically by MiSeq Reporter software version 2.5. The raw Fastq files were trimmed for the presence of Illumina adapter and primer sequences using Cutadapt v3.0 (Martin, 2011).

### Bioinformatics

Quantitative Insights Into Microbial Ecology 2 (QIIME 2) version 2020.8 was used for the analysis of the sequence data (Bolyen et al., 2019). The sequences were imported into QIIME 2 using a Casava 1.8 single-end demultiplexed format. DADA2, a pooled-sample chimera filtering method, was used to denoise the sequences (Callahan et al., 2016). VSEARCH was also used to identify non-16S rRNA gene, chimeric sequence, and open reference clustering of amplicon sequence variants (ASVs) (Rognes et al., 2016). All ASVs were aligned *de novo* using MAFFT and used to construct a phylogenetic tree with FastTree 2 (*via* q2phylogeny) (Price et al., 2010; Katoh and Standley, 2013). Taxonomy was assigned to ASVs using a pretrained Scikit-learn Naive Bayes classifier referencing SILVA database (v. 138) with a 99% identity threshold from 515F/806R (V4) or 388F/518R (V3) region of sequences (Quast et al., 2013; Bokulich et al., 2018; Robeson et al., 2020).

Feature tables that represent the ASV counts for each sample were made in the HDF5-based biological observation matrix (BIOM) format version. Taxa plots were produced using the q2-taxa plug-in. α-Diversity metrics (observed ASVs and Shannon index) and β-diversity metrics (unweighted and weighted UniFrac) were calculated using q2 diversity. The α group significance plugin in Qiime was used to test for differences in α diversity between F1-A and F1-B. The differentially abundant bacterial taxonomies were determined at the genus level using analysis of composition of microbiomes (ANCOM) (Mandal et al., 2015) (*via* q2 composition). PICRUSt2 (phylogenetic investigation of communities by reconstruction of unobserved states) was used to predict the Kyoto Encyclopedia of Genes and Genomes (KEGG) metabolic pathways and COG functional groups from microbiota samples (Douglas et al., 2020). For every individual and KEGG pathway, PICRUSt2 estimates the total gene counts within that pathway (normalized to a relative abundance per pathway).

### Statistical Analysis

All statistical analyses were performed using IBM SPSS 24.0 (IBM SPSS Statistics for Windows, Armonk, NY, United States) with a significance set at a *p* < 0.05. Data were visualized with GraphPad Prism 8.0 (GraphPad Software Inc., La Jolla, CA, United States). To investigate the repeatability of the model seeded with the same batch of inoculum beads, mean daily metabolites concentrations and quantitative microbiota composition in the effluents F1-A and F1-B obtained after initial stabilization were compared among fermentations by Student *t* test after evaluating normal distribution using Shapiro–Wilk test. Comparisons between IR and SSRs measured parameters of F2 and F3 were performed using one-way analysis of variance (Tukey test) after testing for normal distribution using the Shapiro–Wilk test. Data are expressed as means ± standard deviations.

### Data Availability

The sequence data reported in this article have been deposited in the European Nucleotide Archive database (primary accession no. PRJEB47490).

## Results

To establish the *in vitro* chicken cecal microbiota model, physiological parameters such as pH, metabolites, and bacterial composition were analyzed in freshly collected ceca of Cobb-500 broiler chicken.

### Composition of *in vivo* Chicken Cecal Microbiota and Metabolites

**FIGURE 2:**
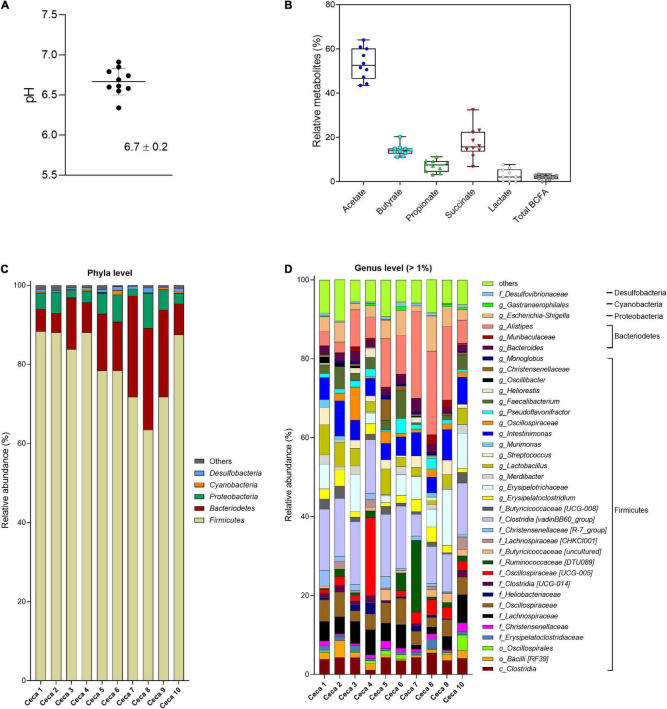
Analysis of cecal content of Cobb-500 broiler chicken (mean ± standard deviation; *n* = 10). **(A)** pH; **(B)** relative metabolite concentrations (%); **(C)** microbial composition obtained by 16S rRNA gene amplicon sequencing and expressed as relative abundance at phylum; and **(D)** genus level. When the genus assignment was not possible, the highest-level taxonomy assignment was shown. Values < 1% are summarized in the group “others”.

Cecal pH in Cobb-500 broiler chicken (*n* = 10) ranged from 6.3 to 6.9 with a mean 6.7 ± 0.2 (Figure 2A). The SCFAs acetate, propionate, and butyrate and the intermediate fermentation metabolites succinate and lactate were detected in all broiler chicken ceca (Figure 2B), whereas formate was below the detection limit of the HPLC method (0.25 μmol/mL). Individual variations of the total metabolite concentration (38.9–50.1 μmol/g cecal content) were observed. Acetate (53.2% ± 7.1%) was the most abundant SCFA, followed by succinate (17.5% ± 7.1%), butyrate (14.2% ± 2.6%), propionate (7.1% ± 2.7%), and lactate (2.8% ± 2.7%), detected at lower levels. BCFAs and valerate were at low levels in the cecal samples. The cecal microbiota was largely dominated by the bacterial phylum Firmicutes (79.9% ± 8.7%), followed by Bacteroidetes (13.9% ± 7.9%), Proteobacteria (4.5% ± 2.3%), and Cyanobacteria (0.4% ± 0.3%) (Figure 2C). Within the Firmicutes phylum, *Clostridia* [vadinBB60_group], unknown *Erysipelotrichaceae*, *Intestinimonas*, unknown *Lachnospiraceae*, and unknown *Oscillospiraceae* were the most abundant taxa in decreasing order. *Alistipes* was the most abundant genus detected within the Bacteroidetes phylum (Figure 2D). The Proteobacteria phylum was dominated by the genus *Escherichia–Shigella*. The α-diversity Shannon index (*H*) ranged from 5.1 to 6.3 (average 5.8 ± 0.4), and the observed ASV richness was on average 128.3 ± 11.2 (Supplementary Figure 1C).

### Designing the Nutritive Medium for *in vitro* Chicken Cecal Fermentation

Different nutritive medium compositions were investigated to improve the similarity between *in vitro* model and chicken cecal microbiota composition and activity (Table 1 and Supplementary Table 1).

#### Adapted mVL-1 and mVL-2 Fermentation Medium for Cecal Inoculum

**FIGURE 3:**
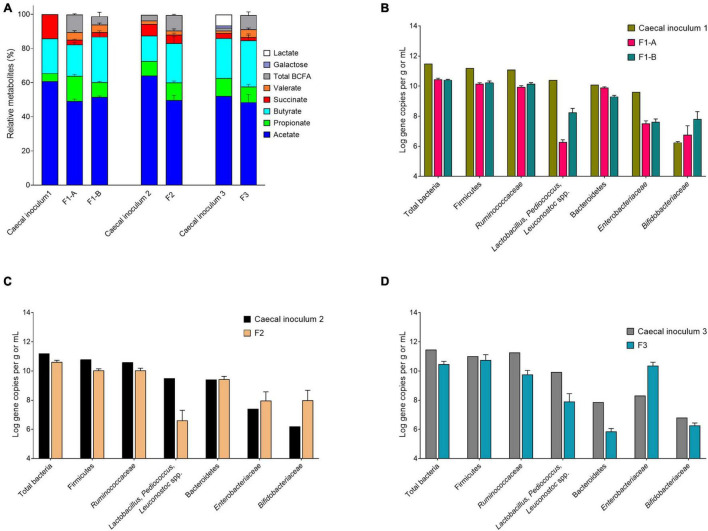
Metabolite and microbial compositions in the cecal inocula and reactor effluents of different fermentations after initial stabilization. **(A)** Relative metabolite ratios and standard deviation (%). **(B–D)** Quantification of bacterial populations by qPCR, expressed as mean ± SD log gene copies/g or mL.

We evaluated the mVL-1 and mVL-2 medium, which were adapted from mVL medium for cecal fermentation, with two *in vitro* fermentation, F1-A and F1-B, inoculated with the same immobilized cecal chicken microbiota. The mVL-2 medium was supplemented with 2.5 g/L FOS to enhance the growth of *Bifidobacterium* and *Lactobacillus* species groups. After bead colonization during two batch fermentations and an initial period of continuous culture, metabolite stability was observed after 6 and 8 days for F1-A and F1-B, respectively (Supplementary Figure 2). mLV-2 nutritive medium resulted in significantly (*p* < 0.05) increased acetate (70.9 ± 2.6 vs. 65.8 ± 4.9 μmol/mL) and butyrate (36.5 ± 2.2 vs. 24.2 ± 1.3 μmol/mL) production, but decreased propionate concentration (11.9 ± 1.1 vs. 19.6 ± 1.8 μmol/mL), compared with mVL-1 (Supplementary Table 3). No significant differences in succinate level and the total metabolite production, expressed by the sum of final and intermediate metabolites in μmol/mL and carbon-mol (C-mol), were detected between both fermentations (Figure 3A and Supplementary Figure 2). Using mVL-1 in F1-A, a 1:1 ratio of propionate and butyrate was observed. However, the inclusion of FOS resulted in a 1:3 ratio in F1-B similar to the ratio of propionate and butyrate (1:4) in the cecal inoculum (Supplementary Table 3). Valerate and BCFAs, which were below the detection limit in the cecal inoculum, were detected in fermentation 1-A (5.8 ± 1.5 and 13.4 ± 0.9 μmol/mL, respectively) and 1-B (6.0 ± 1.2 and 6.7 ± 3.6 μmol/mL, respectively).

The total 16S gene copy numbers measured in F1-A and F1-B were very similar (10.4 ± 0.1 and 10.4 ± 0.0 log gene copies/mL, respectively), and approximately 1 log lower than in the chicken cecum (Figure 3B). The addition of 2.5 g/L FOS in mVL-2 in F1-B resulted in a significant (*p* < 0.05) enrichment of *Lactobacillus–Pediococcus–Leuconostoc* species (+ 2 log gene copies) and *Bifidobacteriaceae* species (+ 1 log gene copies), compared with F1-A (Figure 3B and Supplementary Table 4). However, there was no significant difference (*p* > 0.05) in the other bacterial groups tested by qPCR. Compared with the cecum microbiota composition, all bacterial groups tested *via* qPCR decreased in the model, except *Bifidobacteriaceae* species. The largest decrease observed *in vitro* was for *Lactobacillus–Pediococcus–Leuconostoc* species (−2 log gene copies) and *Enterobacteriaceae* species (−2 log gene copies). In contrast, *Bifidobacteriaceae* species were enriched by 1 log gene copy *in vitro* compared with *in vivo* (Supplementary Table 4).

**FIGURE 4:**
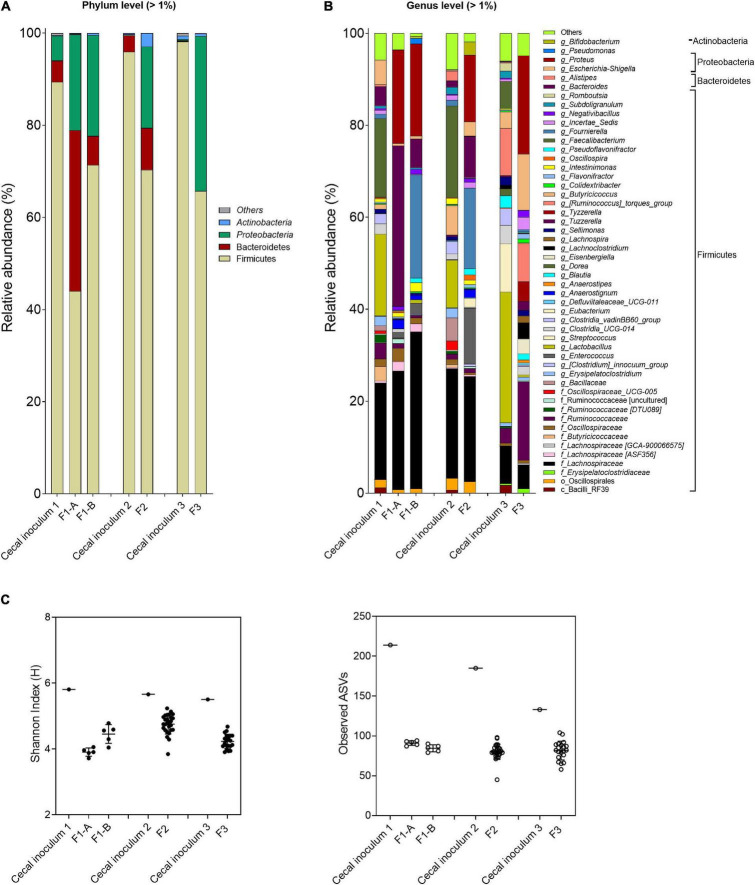
Microbial composition and diversity in cecal inocula and reactor effluents of the different fermentations. **(A,B)** Microbial composition measured by 16S rRNA gene amplicon sequencing, represented by relative abundance at phylum **(A)** and genus levels **(B)**. When assignment at the genus level was not possible, the highest-level taxonomy assignment was shown. Values < 1% are summarized in the group “others”. **(C)** α-Diversity measured by Shannon index and observed ASVs.

The main phyla detected in F1-A and F1-B were Firmicutes, followed by Bacteroidetes and Proteobacteria (Figure 4A). The inclusion of FOS in the nutritive medium (F1-B) enhanced the abundance of Firmicutes. In the absence of FOS (F1-A), the Firmicutes:Bacteroidetes:Proteobacteria ratio was 44:35:21 compared with 71:6:22 and 84:5:5 in F1-B and cecal inoculum, respectively (Supplementary Table 5).

For both fermentations, the abundance of Firmicutes, specifically for *Faecalibacterium* and *Lactobacillus*, decreased, whereas Proteobacteria (genus *Proteus*) increased compared with the cecal inoculum. Specific genera and species were promoted with the FOS-supplemented medium (F1-B) compared with mVL-1 (F1-A) as illustrated using ANCOM analysis (Supplementary Figure 3), with a pronounced increase in *Fournierella* and, to a less extent, in *Pseudomonas* abundance. However, *Clostridium*, *Bacteroides*, and *Bacillus* were established at lower abundance or remained below the detection limit in F1-B compared with fermentation 1-A (Supplementary Figure 3). Similarly, α-diversity values, Shannon indexes (bacterial community evenness), and the number of observed ASVs (bacterial species richness) were not different between F1-A (3.9 ± 0.1 and 91.0 ± 2.9, respectively) and F1-B (4.5 ± 0.3 and 84.4 ± 4.5) and were lower than in the corresponding host cecum (5.8 and 214.0) (Figure 4C).

#### Final mVL-3 Fermentation Medium for Cecal Inoculum

**FIGURE 5:**
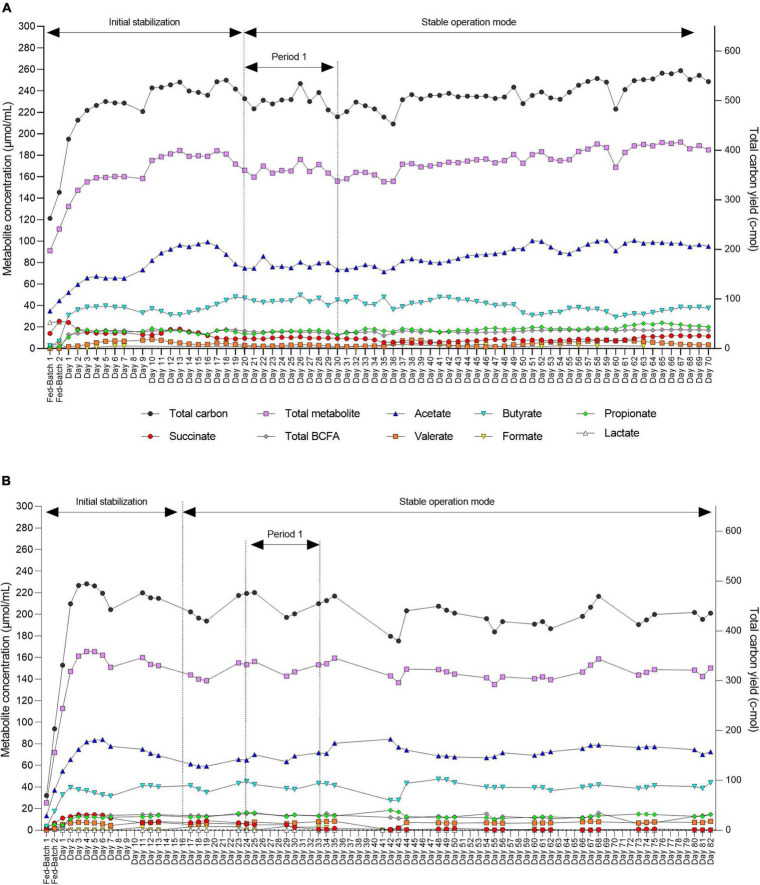
Daily fermentation metabolite concentrations in the effluents of the inoculum reactor (IR) for F2 **(A)** and F3 **(B)** measured by HPLC-RI. End metabolites (acetate, butyrate, propionate, and formate), intermediate metabolite (succinate), BCFAs (isovalerate and isobutyrate), and valerate. Two consecutive batch fermentations were used for bead colonization. Period 1: The period within which the second-stage reactors were connected to IR and stabilize.

The mVL-2 medium was further added with 2.5 g/L pectin to promote Firmicutes and Bacteroidetes (mVL-3 medium) instead of Proteobacteria. Two independent fermentations (F2 and F3) inoculated with different cecal microbiota were used to evaluate the efficacy of mVL-3 fermentation medium for *in vitro* chicken cecal microbiota fermentation. Metabolic stability in the IR of F2 and F3 operated with mVL-3 was reached after approximately 12 days of fermentation as indicated by total metabolite production (Figures 5A,B). A higher total metabolite production was measured in F2 (174.5 ± 10.5 μmol/mL) and F3 (142.1 ± 5.9 μmol/mL) compared with F1-B (137.5 ± 5.9 μmol/mL) (Supplementary Table 3). The use of the mVL-3 nutritive medium resulted in comparable propionate ratios in F2 (12.4% ± 1.2 vs. 9.9%) and F3 (9.2% ± 1.3 vs. 10.4%) to the respective cecal inoculum. However, similar to F1-B, higher levels of butyrate (27.8% ± 4.6 vs. 17.2 and 27.1% ± 2.9 vs. 24.92%) and decreased acetate (59.8% ± 3.8 vs. 72.9 and 48.5% ± 4.6 vs. 52.1%) were detected in F2 and F3 compared with cecal inoculum, respectively.

After an initial stabilization period, we also demonstrated high stability of metabolic activity of the microbiota over 70 and 82 days of continuous operation with constant conditions for both F2 and F3, respectively (Figures 5A,B).

High and stable total 16S rRNA gene copy numbers of *Firmicutes* and *Ruminococcaceae* species were measured in the IR of F2 and F3 after the stabilization period, comparable to the respective cecal inoculum (Figures 3C,D and Supplementary Figure 4). Interestingly, the *in vitro* Bacteroidetes levels (5.9 ± 0.2 log gene copies/mL) were low in F3, in agreement with the low concentrations present in the cecal inoculum (7.9 log gene copies/g). *Bifidobacteriaceae* established at a comparable level in F3 (6.3 ± 0.2 vs. 6.8 log gene copies) but increased in F2 (8.0 ± 0.7 vs. 6.2 log gene copies) compared with their respective cecal inoculum. An increase in *Enterobacteriaceae* species (+1.6 and + 2.0 log gene copies) and a decrease in *Lactobacillus* species (−3.0 and −2.0 log gene copies) was also measured in F2 and F3, respectively, compared with the respective chicken cecum (Figures 3C,D).

The MiSeq data agreed with qPCR analyses, with increased *Escherichia–Shigella* (+ 3 and 12%) and *Proteus* (+ 15 and 21%) and decreased *Faecalibacterium* (−20 and −6%) and *Lactobacillus* (−10 and −28%) in F2 and F3 samples compared with the respective cecal inoculum (Figure 4B and Supplementary Table 5). Similarly, the Shannon index and observed ASVs of F2 (4.8 ± 0.3 and 80.5 ± 9.6, respectively) and F3 (4.2 ± 0.2 and 81.6 ± 11.8) were lower compared with the respective cecal inoculum (5.6 and 185.0; 5.5 and 133.0) (Figure 4C).

### Similarity Between Inoculum Reactors and Second-Stage Reactors of the PolyFermS Fermentation Model of the Chicken Cecal Microbiota

**FIGURE 6:**
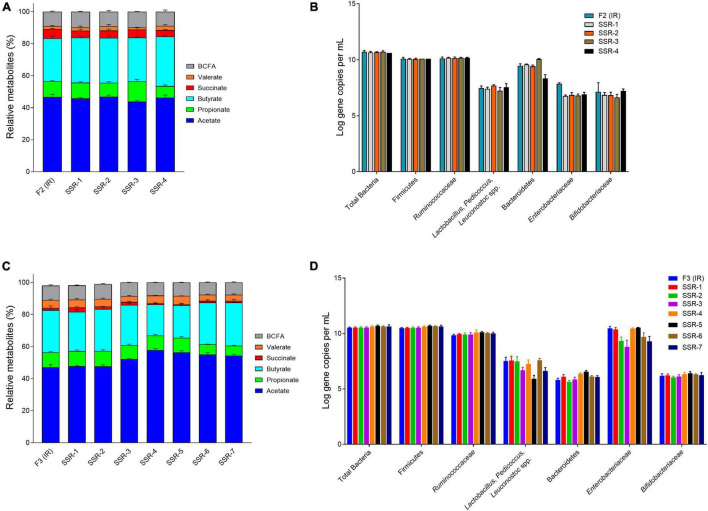
Bacterial activity and composition of inoculum reactor (IR) and second-stage reactors (SSR) after initial stabilization estimated from days 6–10 in period 1. Relative metabolite with standard deviation of F2 **(A)** and F3 **(C)**. Quantification of key bacterial populations by qPCR, expressed as mean ± SD log gene copies/g cecal content or mL effluent of F2 **(B)** and F3 **(D)**.

The PolyFermS platform for intestinal fermentation is designed to allow reproducible and simultaneous testing of different conditions in SSRs, mounted in parallel and continuously inoculated with the microbiota cultivated in IR (Figures 1C,D). Therefore, we evaluated the transfer and reproduction of the chicken cecal microbiota composition and activity in SSRs, by comparison with IR inoculated with the immobilized microbiota (period 1, Figure 5). Metabolic stability in SSRs was reached after 3–6 days of stabilization after connecting to the IR (Supplementary Figures 5A,B). The microbial activity and composition were tested in SSR effluent daily samples between days 6 and 10 and compared with the composition of IR effluents (Figure 6).

In F2, the acetate:propionate:butyrate ratios in the SSR-1 (55:12:33), SSR2 (56:10:34), SSR-3 (52:15:33), and SSR-4 (55:9:36) were comparable IRs (56:12:32) (Figure 6A and Supplementary Table 3). Interestingly, qPCR analyses for all targeted bacterial groups indicated very similar concentrations in IR and SSRs, except for the *Enterobacteriaceae* family, which was 1 log lower (*p* < 0.05) in SSR1-4 compared with IR (Figure 6B).

**FIGURE 7:**
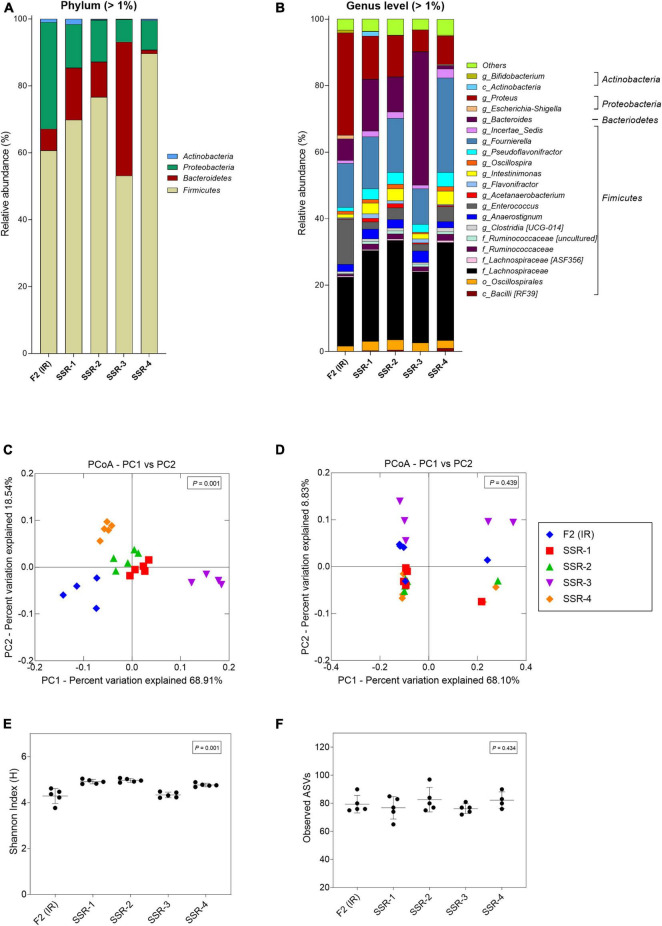
Microbial composition and diversity in the inoculum reactor (IR) and four second-stage reactors (SSR) of F2 after initial stabilization and estimated from days 6–10 in period 1. **(A,B)** Microbial composition by 16S rRNA amplicon sequencing represented by relative abundance at phylum **(A)** and genus levels **(B)**. When the genus assignment was not possible, the highest-level taxonomy assignment was shown. Values < 1% are summarized in the group “others”. **(C,D)** Principal coordinate analysis (PCoA) of reactor microbiota based on weighted **(C)** and unweighted **(D)** UniFrac analysis matrix. α-Diversity measured by **(E)** Shannon index and **(F)** observed ASVs.

From the MiSeq sequencing data, the Firmicutes was the main phylum in all reactors, but variations were observed between IR and SSR1-4 (Figure 7 and Supplementary Table 6). The relative abundance of Proteobacteria decreased in the SSR1-4 (representing 7–13%) compared with IR (24%), whereas the relative abundance of Bacteroidetes showed large variations among SSR1-4, between 1 and 40% compared with 7% in IR, which was confirmed by qPCR data (Figure 7 and Supplementary Table 6). At the genus level, taxonomies with the highest relative abundance at similar levels in the IR and SSR1-4 were unknown *Lachnospiraceae* (21 vs. 21–30%), *Anaerostignum* (2 vs. 2–5%), and *Fournierella* (13 vs. 11–16%), whereas larger differences were observed for *Enterococcus* (14 vs. 2–5%) and *Proteus* (31 vs. 7–13%) (Supplementary Table 6). PCoA analysis on weighted UniFrac distance matrix of microbiota from the SSR1-4 and IR showed clustering based on the reactor type (Figure 7C). However, PCoA biplot on unweighted UniFrac distance analysis reveals the convergence of the microbiota of the F2 (IR) and the SSRs, confirmed by *p* > 0.05 by permutational multivariate analysis of variance PERMANOVA (Figure 7D). There was significant difference (*p* < 0.05) in the α-diversity Shannon indexes among all reactors, SSR1-4 (4.9 ± 0.1, 4.9 ± 0.1, 4.3 ± 0.1, 4.7 ± 0.1) and IR (4.3 ± 0.3) (Figure 7E). However, the observed ASVs between the IR (79.4 ± 6.2) and the SSRs (76.8 ± 7.9, 82.6 ± 8.7, 76.2 ± 3.4, 82.3 ± 5.9) were within the same range (*p* > 0.05) (Figure 7F).

**FIGURE 8:**
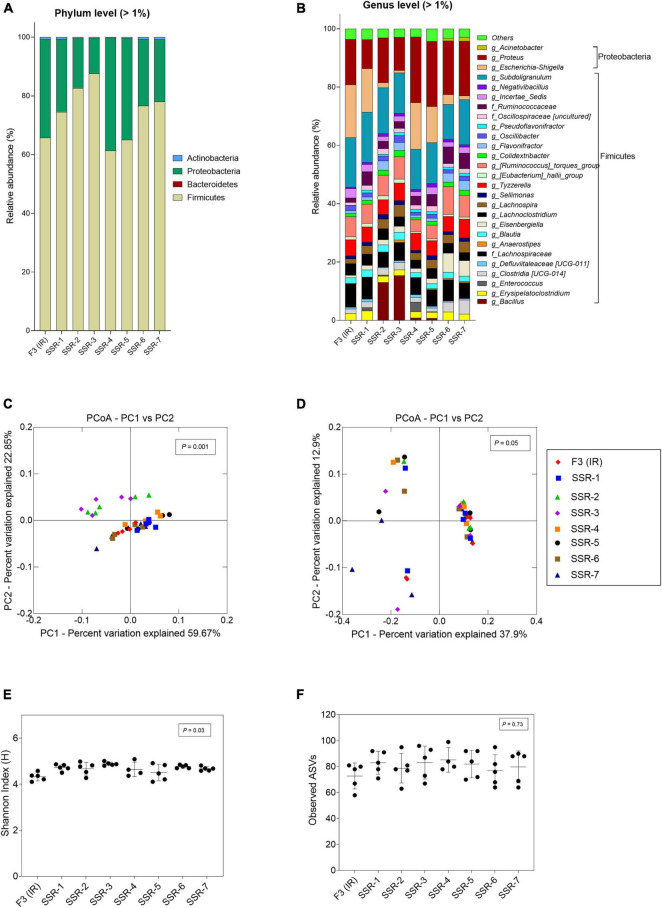
Microbial composition and diversity in the inoculum reactor (IR) and seven second-stage reactors (SSR) of F3 after initial stabilization in period 1. **(A,B)** Microbial composition by 16S rRNA amplicon sequencing represented by relative abundance at phylum **(A)** and genus levels **(B)**. When the genus assignment was not possible, the highest-level taxonomy assignment was shown. Values < 1% are summarized in the group “others”. **(C,D)** Principal coordinate analysis (PCoA) of reactor microbiota based on weighted **(C)** and unweighted **(D)** UniFrac analysis matrix. α-Diversity measured by **(E)** Shannon index and **(F)** observed ASVs.

In F3, the metabolic profile in IR and SSR1-7 was established at similar levels after the initial stabilization period (Supplementary Figure 5B and Figure 6C). Furthermore, qPCR data showed stability of the bacterial groups of IR and SSR1-7, including total bacteria, Firmicutes, Bacteroidetes, *Ruminococcaceae*, and *Bifidobacteriaceae*, with some variations in *Enterobacteriaceae* and *Lactobacillus* (Figure 6D and Supplementary Table 4). Similarly, we observed variations in the relative abundance of the major phyla and genus (Figures 8A,B). PCoA biplot of the microbiota of the IR and SSR1-7 of F3 showed separate clusters and significant difference of the weighted (*p* = 0.001) and unweighted (*p* = 0.05). UniFrac distance matrics by PERMANOVA, respectively (Figures 8C,D). There was a significant difference (*p* < 0.05) in the α-diversity Shannon indexes among IR and SSR1-7 (Figure 8E). However, there was no significant difference (*p* > 0.05) of the observed ASVs between the IR and SSR1-7 of F3 (Figure 8F).

### Functionality of *in vitro* Microbiota in mVL-3 Medium

**FIGURE 9:**
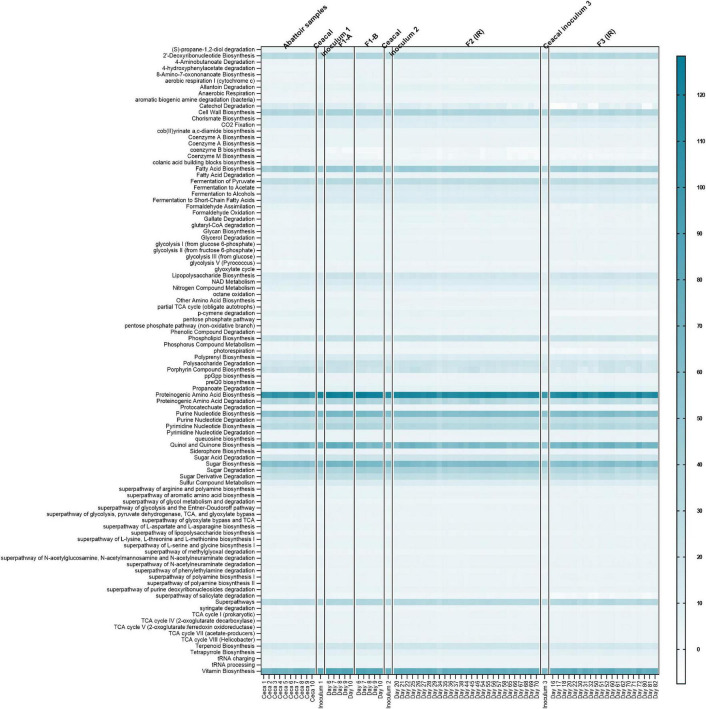
Predictive functional profiling of microbial communities of chicken cecal samples and modeled microbiota of F1-A, F1-B, F2, and F3 using PICRUSt2 analysis. Heatmap depicts the log-transformed gene abundance of microbiota-associated predicted KEGG pathways. Numbers in scale represent log range of gene abundance for this dataset. Darker shades of light blue represent higher relative abundance as indicated in the legend; white color represents absence.

The functional similarity among the cecal fermentation-model-microbiota and cecal samples collected from abattoir as well as those used for inoculum was predicted using the metagenomics imputation method Phylogenetic Investigation of the Communities by Reconstruction of Unobserved States (PICRUSt2) (Langille et al., 2013; Douglas et al., 2020). Conserved metabolic and functional KEGG pathways (level 3) were observed in both microbiota types (Figure 9), indicating a similar microbial functional potential between *in vitro* and the respective cecal microbiota. Furthermore, despite the variations in the metabolic and microbial community profile, there were conserved KEGG functional pathways between the IR and the SSRs of F2 and F3, which were similar to the cecal inoculum (Supplementary Figures 6, 7).

## Discussion

A growing field of research evaluates the impact of nutritional ingredients and supplements on chicken gut health and productivity (Yang et al., 2008; Ricke, 2018; Vermeulen et al., 2018). These studies in chicken are becoming difficult to perform owing to social and economic factors and the call to replace, reduce, and refine (3Rs) in animal testing. The impact of nutrition-related factors on the gut microbial community and functionality can be investigated in a reproducible setting independent of the host in well-designed and validated gut fermentation models operated in conditions closely mimicking the host GIT. Batch fermentation is the most frequently used *in vitro* model for screening of a large number of dietary compounds on the metabolic profile of gut microbiota (Pham and Mohajeri, 2018; Vermeulen et al., 2018). Batch models are fast, inexpensive, easy to operate, and reproducible (Payne et al., 2012). However, batch fermentations are limited in terms of experimental duration (<48–72 h) due to the rapid depletion of substrate, the accumulation of microbial metabolites, and reduction of pH, which can prevent further microbial activity (Payne et al., 2012; Pham and Mohajeri, 2018). In contrast, the more complex continuous culture models are mostly used for long-term in-depth ecological studies and are superior in modeling the dynamic nature of the GIT (Payne et al., 2012). Continuous culture systems allow the adaptation of various parameters, including dilution rate, retention time, pH, and temperature, to meet and maintain optimal growth conditions (Payne et al., 2012; Pham and Mohajeri, 2018). Hence, the choice of a model system to use depends on the research question and the purpose of study (Lacroix et al., 2015).

So far, only a few semicontinuous or continuous intestinal fermentation models inoculated with a free-cell suspension of chicken cecal content have been described. These studies aimed to evaluate the changes in gut bacterial communities tested over limited fermentation periods (7 days) (Yin et al., 2010), the production of volatile fatty acids by chicken gut microbiota (Lei et al., 2012), and the transfer of a multidrug resistance plasmid within microbes in the chicken gut (Card et al., 2017) and to formulate cecal microbial consortia for inoculation of broiler chickens (Gong et al., 2019). The challenge with the use of free-cell suspensions for inoculation is the loss of less dominant or slow-growing bacteria species by rapid washout, the low cell density measured in the fermentation vessel, and the lack of biofilm planktonic growth compared with *in vivo* that can highly impact competition and cross-feeding among bacteria (Payne et al., 2012; Lacroix et al., 2015; Isenring et al., 2021).

In this study, we report for the first time the development of an *in vitro* continuous fermentation model inoculated with immobilized cecal microbiota obtained from healthy 21-day-old Cobb-500 broiler chicken and its validation using the in-depth characterization of microbial metabolites and community. The immobilization aimed to prevent the washout of slow-growing bacteria and reproduce both the sessile and planktonic states of the gut bacteria at high cell densities over a long period. Furthermore, the nutritive medium was investigated, and its composition was adapted to enhance the similarity of microbial composition and activity between modeled and chicken cecal microbiota.

A major challenge in applying highly controlled *in vitro* gut fermentation models is the sampling, handling, and transfer of the microbiota from the host to the bioreactor, including in our case the immobilization step. For accurate modeling of intestinal fermentation, the preservation of bacterial activity, especially the stress-sensitive strict anaerobes in the microbiota of the living host, must be achieved (Lacroix et al., 2015). By using the immobilized cecal microbiota inoculation, a high cell density (approximately 10.5 log cells) and stable *in vitro* fermentation were measured for up to 82 days of continuous operation with acetate, propionate, and butyrate as the major end metabolites and metabolite ratios in agreement with *in vivo* data reported in this and other studies (Walugembe et al., 2015; Dauksiene et al., 2021). In contrast to previous studies and data from cecal samples collected from the abattoir in this study, the total metabolites measured for the cecal samples used to prime up our model appear to be lower. Liao et al. (2020) previously reported that the total metabolites of broiler chicken increase with age. Hence, we speculate that the low total metabolite measured for the cecal content that was used to prime our model was as a result of the age of the chicken (21 days). Higher total metabolite concentrations were measured *in vitro* than *in vivo*, which can be explained by the lack of simulation of metabolite absorption. Hence, the *in vitro* model data better reflect the fermentation capacity of the cecal microbiota, by detecting all produced metabolites.

Valerate and BCFAs (isobutyrate and isovalerate) are products of protein and amino acid fermentation (Macfarlane et al., 1992; Apajalahti and Vienola, 2016; Ma et al., 2017). In our model, valerate and BCFAs were detected at higher levels compared with low amounts or no detection in the chicken cecal content, in agreement with previous studies of cecal digesta of broiler chicken (Rehman et al., 2008; Tiihonen et al., 2010; Dauksiene et al., 2021). We speculate that the high level of proteins in our fermentation medium, the long retention time of 24 h with complete fermentation of degraded carbohydrates, and the lack of absorption contributed to the observed accumulation of valerate and BCFA in our model. This is supported by PICRUSt2 analysis where the proteinogenic amino acid biosynthesis pathway dominated the functional potential of the model and cecal microbiota. Furthermore, bacteria of the phylum Proteobacteria are known for their potential to produce BCFAs (Sridharan et al., 2014; Kaur et al., 2017). Interestingly, a significantly high relative abundance of Proteobacteria was measured in the model compared with the cecal inoculum, which might also contribute to the increased synthesis of BCFAs. Card et al. (2017) previously observed an increase in Proteobacteria in a semicontinuous *in vitro* model of the chicken cecal microbiota. Proteobacteria often bloom in *in vitro* continuous gut fermentation models because of their competitive advantage during the initial colonization period and their high tolerance to environmental stresses and adaptation to the microbial community and metabolism in model reactor conditions (Van Den Abbeele et al., 2010; Tanner et al., 2014a).

Here, there was an overall shift in microbial composition and a decrease in diversity *in vitro* compared with *in vivo* cecal inoculum as measured by qPCR and 16S rRNA sequencing. Such decrease was also observed in other *in vitro* fermentation models from chicken, humans (infants, adult, and elderly), and swine (Van Den Abbeele et al., 2010; Tanner et al., 2014b; McDonald et al., 2015; Card et al., 2017; Oost et al., 2021). Indeed, *in vitro* models cannot simulate all conditions encountered in the host, which are highly individual and not well known or microbiota–host complex interactions that cannot be mimicked, such as the immune response, hormone and digestive secretions, adsorption, and peristaltic movement, all of which influences microbial diversity (Lacroix et al., 2015). The fixed well-controlled *in vitro* conditions may result in a loss of redundant species or species thriving on specific host secretions. This may partly explain why the prevalent chicken cecal bacterial genera *Faecalibacterium* and *Lactobacillus* were less maintained in our *in vitro* fermentation model. *Faecalibacterium* species are a significant representative of the Firmicutes phylum, an acetate-consuming and butyrate-producing bacterium and highly prevalent in chicken, resulting in better feed conversion efficiency (Stanley et al., 2016; Wang et al., 2016). 16S rRNA gene-based analysis of mucosa-associated bacterial communities in the chicken GIT revealed that *Faecalibacterium* species are dominant in the mucosal lining of chicken ceca (Gong et al., 2019). In addition to locally grown microbes, the cecum harbors transient bacteria that are shed from the upper GIT and ingested microbes in the feed and environment because of the coprophagic behavior of the chicken. *Lactobacillus* species are known colonizers of the chicken upper GIT, specifically the crop where they form biofilms at the non-glandular squamous epithelium and are commonly encountered in the ceca due to shedding of the crop biofilm and consequently being transferred to the lower GIT (Fuller and Brooker, 1974; Guan et al., 2003; Abbas Hilmi et al., 2007). The continuous supply of microbes, especially lactobacilli from the crop, is not simulated in our model; in any other model of chicken, it may explain the progressive loss of this bacterial group in the reactor effluents.

Although certain microbial taxa were reduced, taxa within the chicken ceca bacterial families that were preserved in our *in vitro* model of the chicken ceca belong to relevant functional groups such as primary fibrolytic (*Bacteroides*, *Bifidobacteriaceae*, *Ruminococcaceae*), glycolytic (*Enterococcus*), mucolytic (*Bacteroides*), proteolytic (*Bacteroides*), and secondary butyrate-producing, acetate-utilizing, and propionate-producing communities (*Lachnospiraceae*) (Chassard and Lacroix, 2013; Medvecky et al., 2018). This was supported by qualitative assessment of microbial function by PICRUSt2 analysis, which showed that despite reduced diversity, functional redundancy preserved the relative abundance profile of major KEGG categories in the fermentation model. These bacterial groups were significantly decreased in the *in vitro* model of the chicken cecal microbiota developed by Card et al. (2017).

Reproducible microbiota composition and metabolic activity are required to accurately compare the effects of different conditions (e.g., nutritional ingredients and supplements), gain reliable information, and perform a sensitive comparison. In this study, we used a constant 5% inoculation rate from IR to subsequent SSR reactors to allow reproducing a similar and parallel evolving self-contained ecosystem in IR and the SSRs. However, the observed variations in the microbiota composition in the SSRs in comparison with the IR could be partly due to the limited accuracy of the pumps that are required to inoculate all SSRs by the effluent of the IR, which is heterogeneous, containing particulates, as a result of the retention time (24 h) adopted in the model operation. Indeed, we reported a highly reproducible microbiota composition and activity in SSRs of different model hosts using the same platform and shorter retention time, including swine (RT = 9 h), human infants (RT = 5 h), and human elderly (RT = 7.7 h) (Tanner et al., 2014a; Fehlbaum et al., 2015; Pham et al., 2019). To further improve the reproducibility and maintain some of the abundant genera of cecum inoculum such as the *Faecalibacterium* in our PolyFermS model, different options may be tested such as using pumps with higher accuracy, decreasing medium particulates that may have interfered with the pump’s accuracy, using larger connecting tubing, and/or using a shorter mean retention time (12 h). Nevertheless, the difference in the microbiota composition did not lead to a difference in metabolic functions in the SSRs and IR of our PolyFermS model. The functional redundancy within the microbiota may have contributed to the high similarities observed for the metabolite production in the SSRs and IR, as previously reported (Moya and Ferrer, 2016; Heintz-Buschart and Wilmes, 2018; Hankel et al., 2019). This shows that the functionality of the chicken cecal microbiota is well preserved *in vitro* and is despite the compositional difference between the SSR and IR. Hence, our model can be used to assess the impact of abiotic or biotic treatments on the cecal microbiota functionality *ex vivo*.

## Conclusion

The PolyFermS model of the chicken cecal microbiota fermentation described here provides a close reproduction of the composition and activity of the chicken cecal microbiota *in vivo*. The modified VL media supplemented with both FOS and citrus pectin sustained the mimicking of high cell density akin to *in vivo*, as well as bacterial community profiles and metabolite productions reflecting those reported for chicken ceca *in vivo*. This medium would also be suitable for simple batch fermentation of the chicken cecal microbiota. Our new continuous gut model may be particularly useful for detailed functional characterization of chicken cecal microbiota and allow the accurate screening of the impact of several conditions and select factors, including nutritional factors (prebiotics, probiotics, and other dietary additives) on the chicken cecal microbiota, before *in vivo* testing. However, *in vitro* digestion of these nutritional factors will be needed prior to testing in the model to account for upper GIT adsorption and degradation of these compounds.

## Data Availability Statement

The datasets presented in this study can be found in online repositories. The names of the repository/repositories and accession number(s) can be found in the article/Supplementary Material.

## Author Contributions

PTA, AGr, AGe, and CL designed the study. PTA, AP, KB, and AGr conducted the experiments. PTA, AP, KB, AGr, and CL analyzed the data. PTA, AGr, AGe, CS, RS, and CL drafted the manuscript. All authors read and approved the final manuscript.

## Conflict of Interest

The authors declare that the research was conducted in the absence of any commercial or financial relationships that could be construed as a potential conflict of interest.

## Publisher’s Note

All claims expressed in this article are solely those of the authors and do not necessarily represent those of their affiliated organizations, or those of the publisher, the editors and the reviewers. Any product that may be evaluated in this article, or claim that may be made by its manufacturer, is not guaranteed or endorsed by the publisher.
