# Supplementary material for: In vitro Modeling of Chicken Cecal Microbiota Ecology and Metabolism Using the PolyFermS Platform
Source: Front Microbiol. 2021 Dec 20;12:780092. doi: 10.3389/fmicb.2021.780092 (PMC8721126; doi:10.3389/fmicb.2021.780092)
Supplement: Supplementary file 1 [file Data_Sheet_1.docx]

**SUPPLEMENTARY MATERIAL**

***In vitro* Modeling of Chicken Cecal Microbiota Ecology and Metabolism Using the PolyFermS Platform**

Paul Tetteh Asare^1,3^, Anna Greppi^1^, Alessia Pennacchia^1^, Katharina Brenig^1^, Annelies Geirnaert^1^, Clarissa Schwab^1,4^, Roger Stephan^2^, Christophe Lacroix^1*^

^1^Laboratory of Food Biotechnology, Institute of Food, Nutrition and Health, ETH Zürich, Zürich, Switzerland.

^2^Institute for Food Hygiene and Safety, University of Zürich, Zürich, Switzerland.

^3^Present address, Department of Fundamental Microbiology, University of Lausanne, Lausanne, Switzerland.

^4^Present address, Department Biological and Chemical Engineering, Aarhus University, Aarhus, Denmark.

*Correspondence:

Christophe Lacroix

[christophe.lacroix@hest.ethz.ch](mailto:christophe.lacroix@hest.ethz.ch)


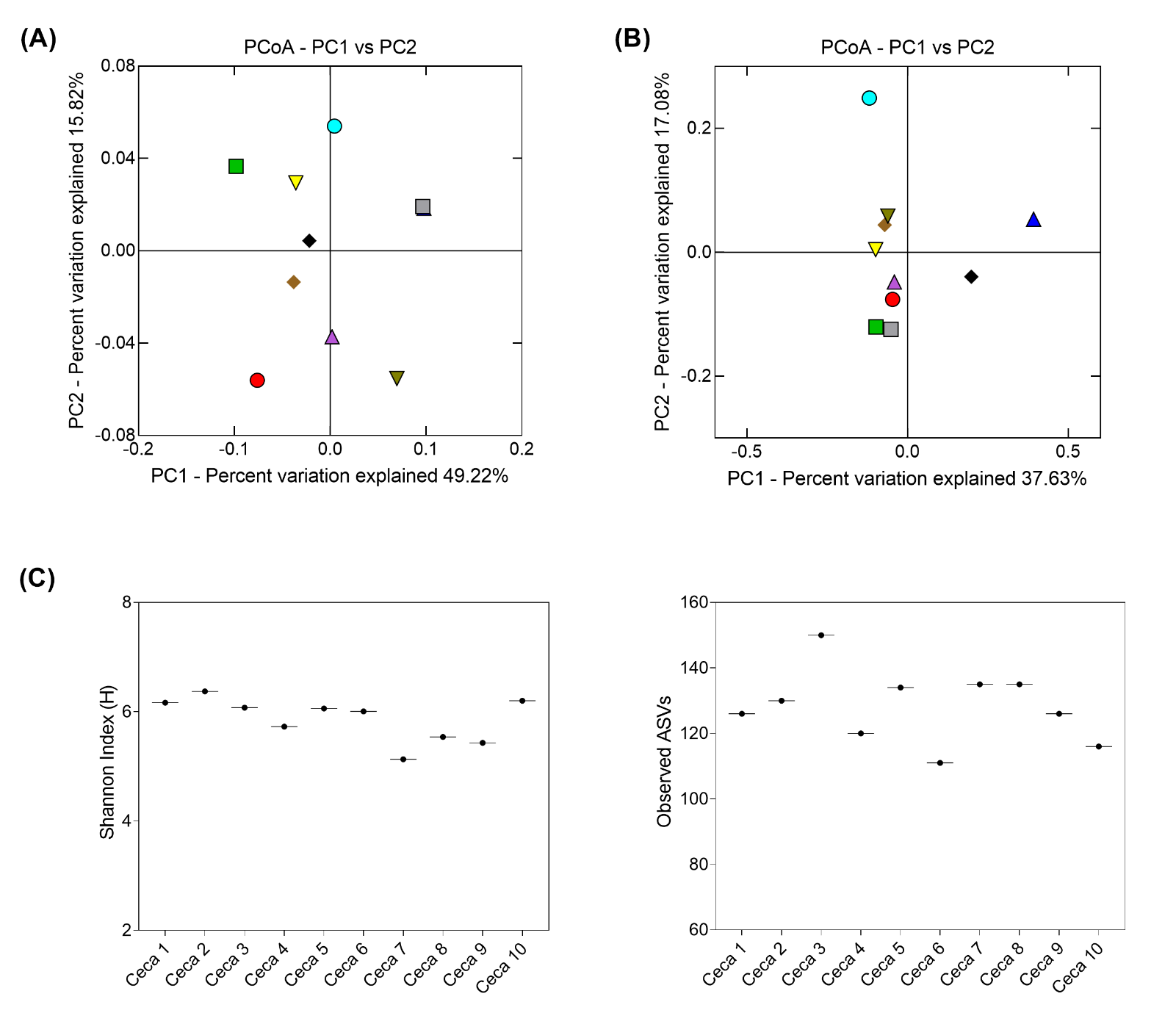


**Supplementary Figure 1.** Individual variations among broiler chicken ceca microbial communities (n = 10). PCoA analysis showing cecal microbiota from Cobb-500 broiler chicken at slaughter (day 35) based on weighted **(A)**, and unweighted Unifrac distance **(B).**  (**C**) Alpha diversity measured by Shannon index and Observed ASVs.


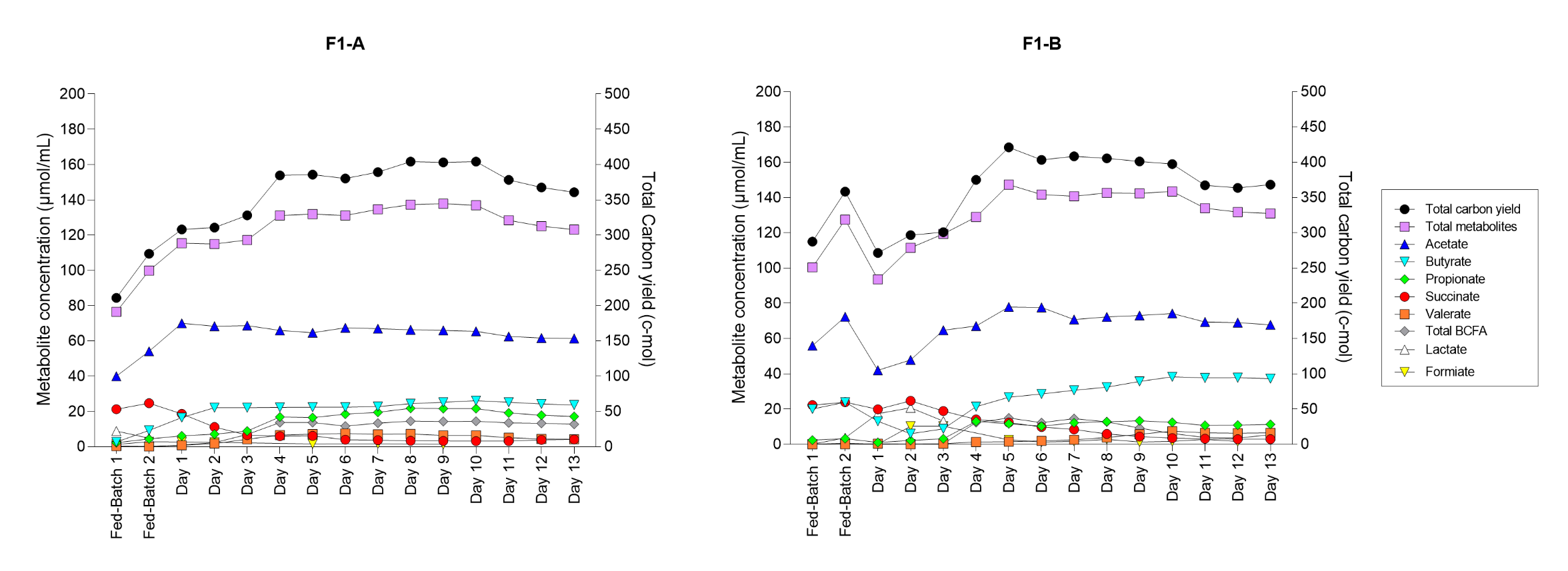


**Supplementary Figure 2.** Daily fermentation metabolite concentrations in reactor effluent for F1-A and F-B measured by HPLC-RI. End metabolites (acetate, propionate, butyrate and formate), intermediate metabolite (lactate, succinate), branched short-chain fatty acids (isobutyrate, isovalerate) and valerate on left y-axis. Total carbon yield on right y-axis.

Log_2_ fold change

**
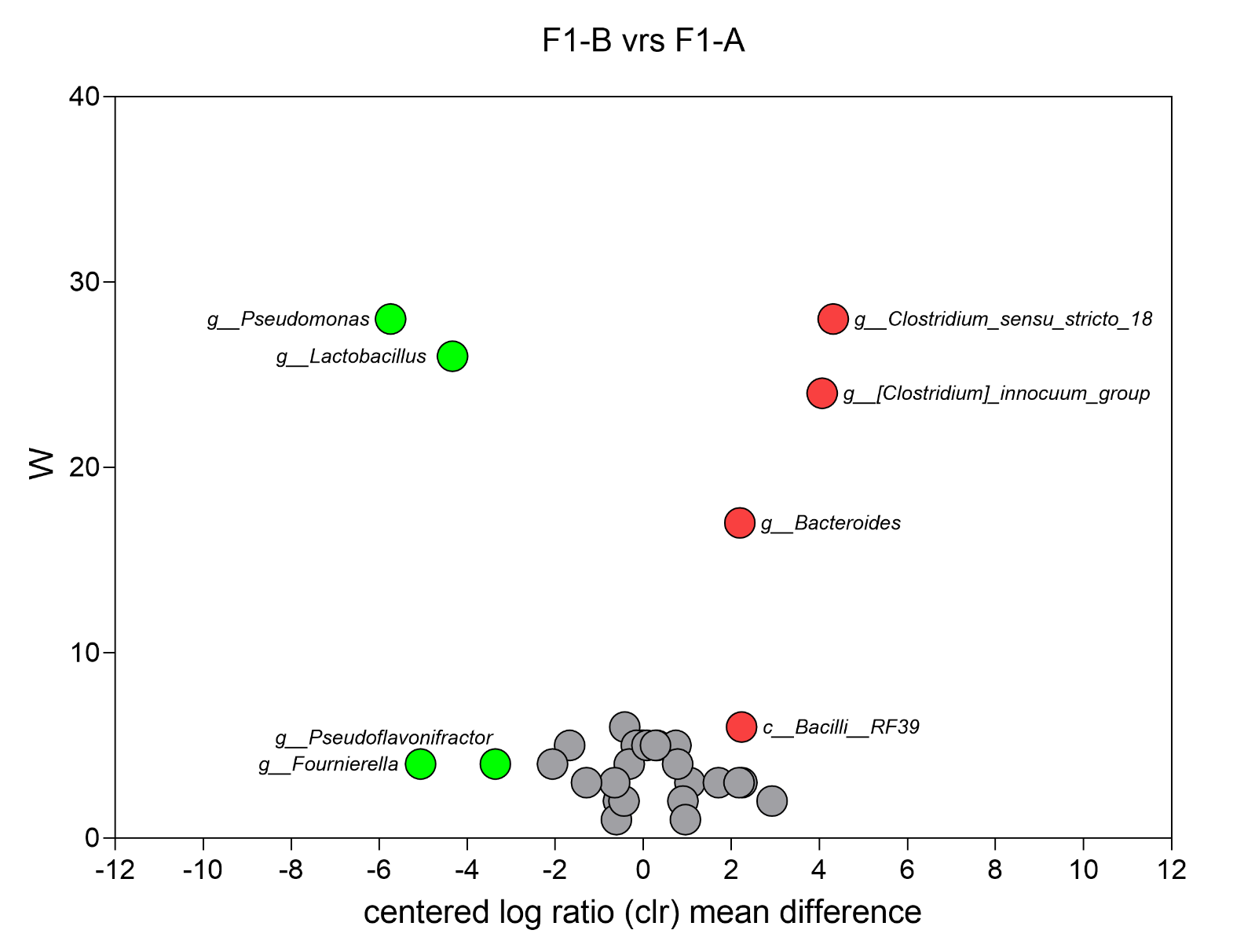
**

**Supplementary Figure 3. Impact of FOS supplementation:** ANCOM volcano plot illustrating the bacterial taxa that were significantly enriched (green) and reduced (red) in the reactor effluent of fermentation 1-B (+FOS) versus reactor effluent of F1-A (-FOS). For ANCOM analysis, the clr (centered log ratio) transformed ASVs at the genus level that was modified to adjust 0 values to 1 was used. The W value represents the number of times of the null-hypothesis (the average abundance of a given species in a group is equal to that in the other group) was rejected for a given species. The x-axis value represents the clr transformed mean difference in abundance of a given species between the F1-A and F1-B. A positive x-axis means a species is abundant in F1-A and a negative x-axis value means a species is abundant in F1-B. Only species with reject null-hypothesis >95% are labelled


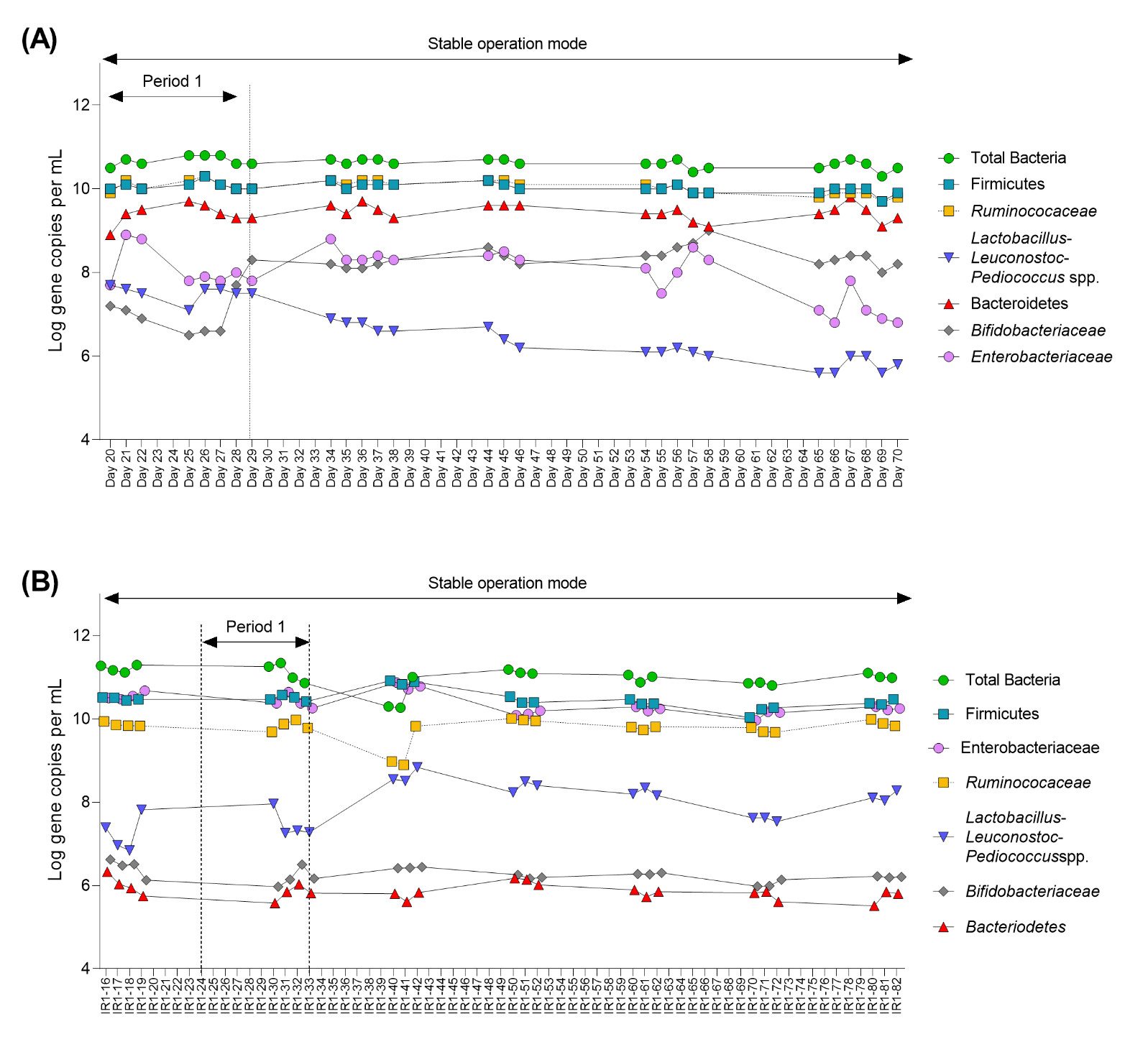


**Supplementary Figure 4.** Quantification of key bacterial populations (16S rRNA gene copy numbers) in the effluent of F2 (**A**) and F3 (**B**) during stable operation by qPCR. Data were expressed as average from technical duplicates


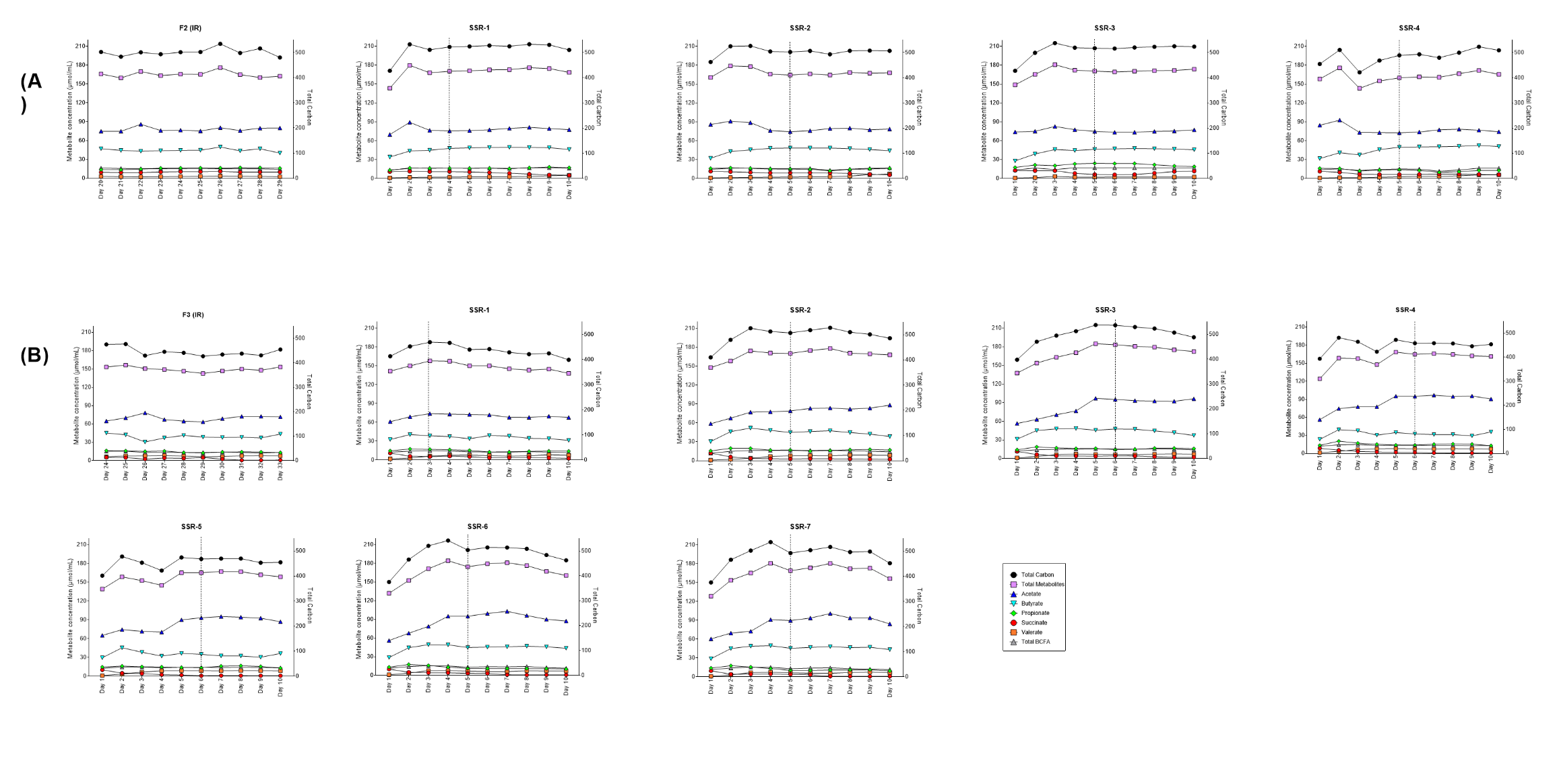


**Supplementary Figure 5.** Daily fermentation metabolite concentrations in reactor effluents of (**A**) F2 (IR) and the four second-stage reactors and (**B**) F3 (IR) and the seven second-stage reactors during period 1 measured by HPLC-RI. The corresponding IR days for the SSRs are plotted. End metabolites (acetate, butyrate, propionate and formate), intermediate metabolite (succinate), BCFAs (isovalerate and isobutyrate) and valerate on left y-axis. Total carbon yield on right y-axis. Horizontal dash line indicates point of metabolic stabilization where the individual variations of metabolites is lower than 10% in the same reactor.


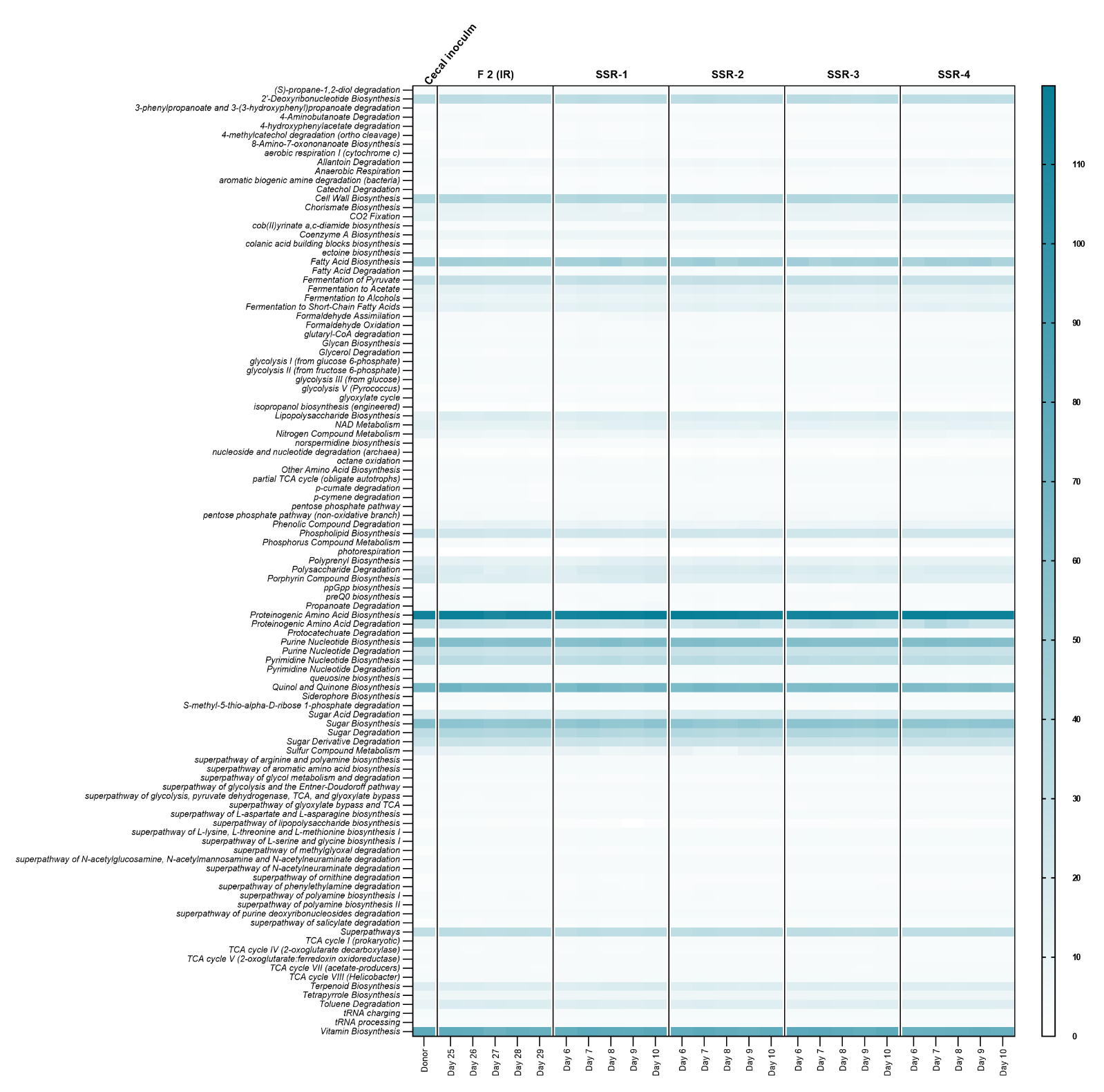


**Supplementary Figure 6.** Predictive functional profiling of microbial communities of cecal inoculum 2 and effluent of F2 (IR) and four second-stage reactors (SSRs) after initial stabilisation in **Period 1** by PICRUSt2. Heatmap depicting the log-transformed gene abundance of microbiota-associated predicted KEGG pathways. Numbers in scale represent log range of gene abundance for this dataset. Darker shades of light blue represent higher relative abundance as indicated in the legend; white colour represents absence.


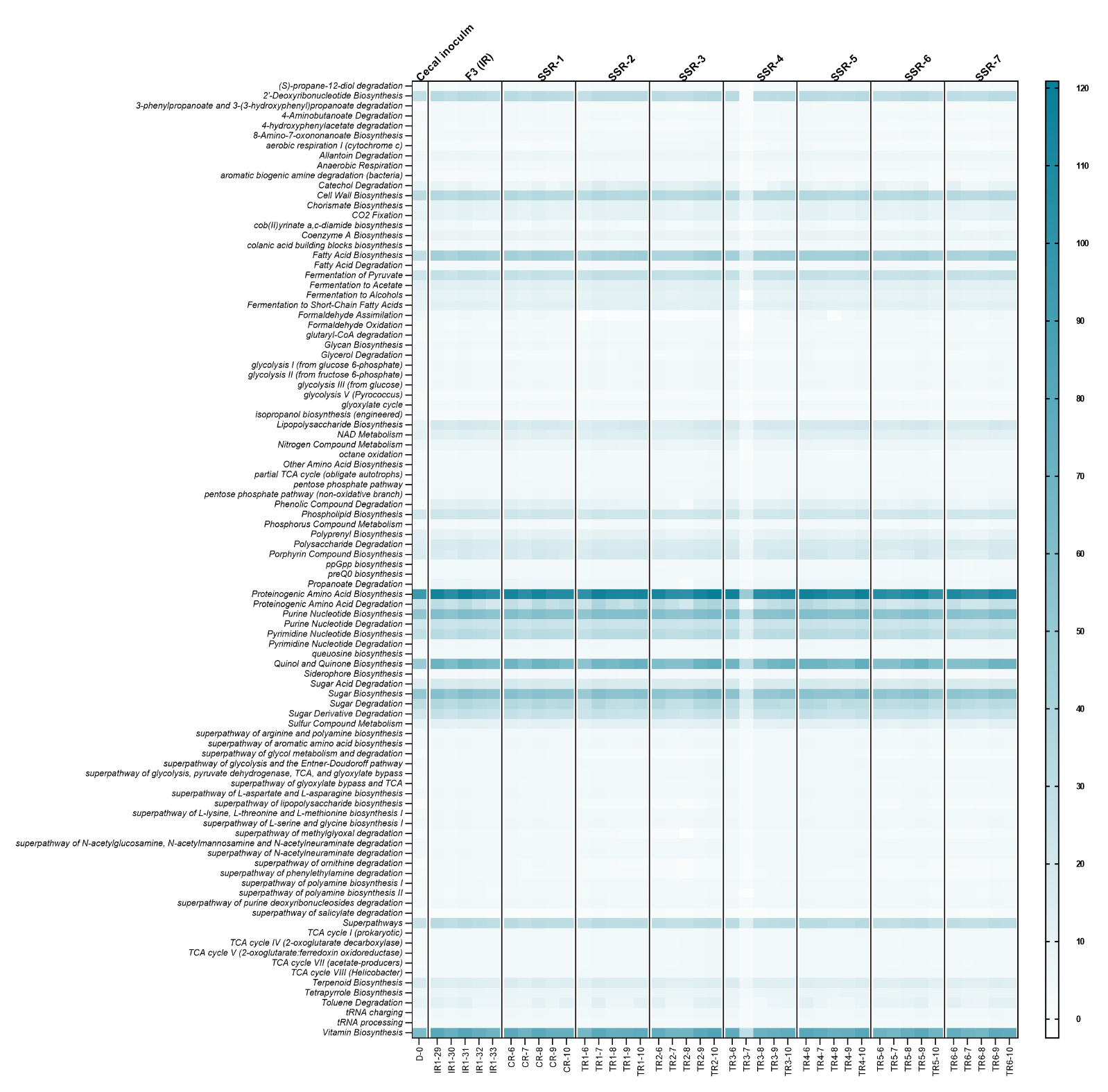


**Supplementary Figure 7.** Predictive functional profiling of microbial communities of cecal inoculum 3 and effluent of F3 (IR) and seven second-stage reactors (SSRs) after initial stabilisation in **Period 1** by PICRUSt2. Heatmap depicting the log-transformed gene abundance of microbiota-associated predicted KEGG pathways. Numbers in scale represent log range of gene abundance for this dataset. Darker shades of light blue represent higher relative abundance as indicated in the legend; white colour represents absence.

|  | **mVL-1** | **mVL-2** | **mVL-3** |
| --- | --- | --- | --- |
| **Constituent** | **g/L** | **g/L** | **g/L** |
| Fructooligosaccharides (FOS) | 0 | 2.5 | 2.5 |
| Pectin (Citrus) | 0 | 0 | 2.5 |
| Beef extract | 2.4 | 2.4 | 2.4 |
| Yeast extract | 5.0 | 5.0 | 5.0 |
| Maltodextrin | 2.5 | 2.5 | 2.5 |
| Tryptose | 10.0 | 10.0 | 10.0 |
| L-cysteine hydrochloride | 0.8 | 0.8 | 0.8 |
| NaCl | 5.0 | 5.0 | 5.0 |
| Mucin | 2.0 | 2.0 | 2.0 |
| Uric acid | 0.7 | 0.7 | 0.7 |
| Tween 80 | 1 mL | 1 mL | 1 mL |
| Bile salts | 0.4 | 0.4 | 0.4 |
| KH_2_PO_4_ | 0.5 | 0.5 | 0.5 |
| NaHCO_3_ | 1.5 | 1.5 | 1.5 |
| KCl | 4.5 | 4.5 | 4.5 |
| MgSO_4_ anhydrous | 0.6 | 0.6 | 0.6 |
| CaCl_2_ x 2H_2_O | 0.1 | 0.1 | 0.1 |
| MnCl_2_ x 4H_2_O | 0.2 | 0.2 | 0.2 |
| FeSO_4_ x 7H_2_O | 0.005 | 0.005 | 0.005 |
| Hemin solution | 0.05 | 0.05 | 0.05 |
| Vitamin solution | 1 mL | 1 mL | 1 mL |

**Supplementary Table 1**. Composition of modified Vainde Levure medium

**Supplementary Table 2**. Primers for qPCR detection of specific bacterial groups

| **Primer** | **Sequence 5’ – 3’** | **Target** | **Reference** |
| --- | --- | --- | --- |
| Eub338F  Eub518R | ACT CCT ACG GGA GGC AGC AG  ATT ACC GCG GCTVGCT GG | Total bacteria | Guo *et al.* (2008) |
| Firm 934F  Firm 1060R | GGA GYA TGT GGT TTA ATT CGA AGC A  AGC TGA CGA CAA CCA TGC AC | Firmicutes | Guo *et al.* (2008) |
| Bac303F  Bfr-Femrev | GAA GGT CCC CCA CAT TG  CGC KAC TTG GCT GGT TCA G | Bacteroidetes | Ramirez-Farias *et al.* (2009) |
| RumiF  RumiR | ACTGAGAGGTTGAACGGCCA  CCTTTACACCCAGTAAWTCCGGA | *Ruminococcaceae* | Garcia-Mazcorro *et al*. (2012) |
| F_Lacto 05  R_Lacto 04 | AGC AGT AGG GAA TCT TCC A  CGC CAC TGG TGT TCY TCC ATA TA | *Lactobacillus-Leuconostoc-Pediococcus* spp. | Furet *et al.* (2009) |
| Bifi_F  Bifi_R | TCG CGT CYG GTG TGA AAG  CCA CAT CCA GCR TCC AC | *Bidfidobacteriaceae* | Meimandipour *et al.* (2010) |
| Eco1457F  Eco1652R | CAT TGA CGT TAC CCG CAG AAG AAG CCTC TAC GAG ACT CAA GCT TGC | *Enterobacteriaceae* | Bartosch *et al*. (2004) |
| pduC_F  pduC_R | CCTGAAGTAAAYCGCATCTT  GAAACYATTTCAGTTTATGG | Reuterin-producing *L. reuteri* | Walter *et al*. (2011) |

**Supplementary Table 3**. Mean metabolite concentrations with standard deviation in reactor effluents after initial the stabilisation phase and in chicken cecal content

|  | **Days** |  | **Concentration (µmol/mL)** | | | | | | |  | **Ratios of Total SCTAs (%)** | | |
| --- | --- | --- | --- | --- | --- | --- | --- | --- | --- | --- | --- | --- | --- |
|  |  |  | **Acetate** | **Butyrate** | **Propionate** | **Succinate** | **Valerate** | **Total BCFAs** | **Total metabolites** |  | **Acetate** | **Propionate** | **Butyrate** |
| Cecal inoculum 1^*^ |  |  | 11.3 | 3.8 | 0.9 | 2.7 | ND | nd | 18.6 |  | 70.6 | 5.6 | 23.8 |
| **F1-A** | D6-13 |  | 65.8 ± 4.9^A^ | 24.2 ± 1.3^a^ | 19.6 ± 1.8^A^ | 3.5 ± 0.4^a^ | 5.8 ± 1.5^A^ | 13.4 ± 0.9^a^ | 131.8 ± 5.8^A^ |  | 59.6 ± 1.5^a^ | 18.0 ± 1.0^A^ | 22.3 ± 1.1^a^ |
| **F1-B** | D8-13 |  | 70.9 ± 2.6^B^ | 36.5 ± 2.2^b^ | 11.9 ± 1.1^B^ | 3.8 ± 1.2^a^ | 6.0 ± 1.2^A^ | 6.7 ± 3.6^b^ | 137.5 ± 5.9^A^ |  | 59.5 ± 1.2^a^ | 10.0 ± 0.8^B^ | 30.6 ± 1.8^b^ |
| Cecal inoculum 2^*^ |  |  | 19.9 | 4.7 | 2.7 | 2.2 | 0.6 | 1.1 | 31.2 |  | 72.9 | 9.9 | 17.2 |
| **F2** (IR) | D20 - 70 |  | 86.9 ± 9.4 | 40.0 ± 5.4 | 18.0 ± 2.5 | 8.6 ± 1.9 | 4.2 ± 2.0 | 16.0 ± 1.3 | 174.5 ± 10.5 |  | 59.8 ± 3.8 | 12.4 ± 1.2 | 27.8 ± 4.6 |
| **F2** (IR) | D25-29 |  | 78.2 ± 2.5^AB^ | 44.9 ± 3.6^a^ | 16.5 ± 0.4^A^ | 10.0 ± 0.5^a^ | 3.1 ± 0.5^AB^ | 15.1 ± 0.5 ^ab^ | 168.2 ± 5.4^AB^ |  | 56.0 ± 1.6^a^ | 11.8 ± 0.5^A^ | 32.1 ± 1.7^a^ |
| SSR-1 | D6-10 |  | 79.1 ± 1.6^A^ | 48.7 ± 1.4^bc^ | 17.0 ± 1.0^A^ | 7.3 ± 1.8^ab^ | 3.9 ± 1.1^AB^ | 16.7 ± 0.4^a^ | 175.8 ± 2.8^A^ |  | 54.6 ± 0.5^a^ | 11.7 ± 0.7^A^ | 33.6 ± 0.7^a^ |
| SSR-2 | D6-10 |  | 78.2 ± 1.6^AB^ | 46.8 ± 1.8^ab^ | 14.4 ± 1.4^AC^ | 7.6 ± 1.5^ab^ | 4.4 ± 2.0^AB^ | 15.3 ± 1.5^ab^ | 166.8 ± 1.6^AB^ |  | 56.1 ± 0.8^a^ | 10.3 ± 1.1^A^ | 33.6 ± 1.2^a^ |
| SSR-3 | D6-10 |  | 74.9 ± 1.6^B^ | 46.9 ± 0.8^ab^ | 21.6 ± 2.2^B^ | 8.7 ± 2.5^ab^ | 2.3 ± 0.1^A^ | 16.8 ± 0.2^a^ | 171.3 ± 1.6^AB^ |  | 52.3 ± 1.9^b^ | 15.0 ± 1.4^B^ | 32.7 ± 0.3^a^ |
| SSR-4 | D6-10 |  | 76.1 ± 1.9^AB^ | 51.0 ± 0.9^c^ | 11.8 ± 1.4^C^ | 6.6 ± 0.4^b^ | 4.5 ± 1.1^B^ | 14.5 ± 1.1^b^ | 165.2 ± 4.4^B^ |  | 54.6 ± 1.2^a^ | 8.5 ± 1.0^C^ | 36.7 ± 0.2^b^ |
| Cecal inoculum 3* |  |  | 11.4 | 5.4 | 2.3 | 0.64 | 0.36 | 0.34 | 26.9 |  | 52.1 | 10.4 | 24.92 |
| **F3 (IR)** | D16-82 |  | 70.8 ± 6.6 | 39.9 ± 4.3 | 13.3 ± 1.7 | 2.4 ± 2.4 | 6.8 ± 1.4 | 12.4 ± 2.6 | 142.1 ± 5.9 |  | 48.5 ± 4.6 | 9.2 ± 1.3 | 27.1 ± 2.9 |
| **F3 (IR)** | D29-33 |  | 69.7 ± 3.9^C^ | 39.1 ± 2.5^b^ | 13.7 ± 0.8^B^ | 1.9 ± 1.9^abc^ | 7.4 ± 0.9^BCD^ | 13.3 ± 0.6^b^ | 148.1 ± 3.9^D^ |  | 47.1 ± 1.8^e^ | 9.2 ± 0.5^A^ | 26.4 ± 1.3^ab^ |
| SSR-1 | D6-10 |  | 70.1 ± 2.2^C^ | 35.9 ± 2.4^bc^ | 13.7 ± 1.1^AB^ | 4.1 ± 0.7^a^ | 7.3 ± 0.8^BCD^ | 13.1 ± 0.2^b^ | 146.8 ± 3.2^D^ |  | 47.7 ± 0.6^e^ | 9.4 ± 0.7^A^ | 24.5 ± 1.6^c^ |
| SSR-2 | D6-10 |  | 82.1 ± 1.9^B^ | 45.1 ± 2.1^a^ | 16.6 ± 0.9^A^ | 2.8 ± 0.2^abc^ | 8.3 ± 0.5^AB^ | 15.9 ± 0.7^a^ | 172.6 ± 3.7^BC^ |  | 47.6 ± 1.1^e^ | 9.6 ± 0.7^A^ | 26.1 ± 0.7^ab^ |
| SSR-3 | D6-10 |  | 94.0 ± 1.9^A^ | 45.3 ± 2.4^a^ | 15.8 ± 1.1^A^ | 3.4 ± 0.9^ab^ | 6.7 ± 0.7^CD^ | 15.4 ± 0.3^a^ | 180.6 ± 3.8^A^ |  | 52.1 ± 0.5^d^ | 8.7 ± 0.7^A^ | 25.1 ± 0.9^ab^ |
| SSR-4 | D6-10 |  | 95.6 ± 0.9^A^ | 31.9 ± 1.9^c^ | 14.9 ± 0.9^AB^ | 1.3 ± 0.6^bc^ | 7.9 ± 0.3^ABC^ | 13.6 ± 0.3^b^ | 165.3 ± 2.3^C^ |  | 57.8 ± 0.8^a^ | 9.1 ± 0.6^A^ | 19.3 ± 0.9^c^ |
| SSR-5 | D6-10 |  | 93.0 ± 1.9^A^ | 33.3 ± 2.4^c^ | 15.2 ± 1.3^AB^ | 1.1 ± 0.5^c^ | 8.7 ± 0.2^A^ | 14.1 ± 0.1^b^ | 165.3 ± 2.1^C^ |  | 56.3 ± 1.1^ab^ | 9.2 ± 0.8^A^ | 20.1 ± 1.4^c^ |
| SSR-6 | D6-10 |  | 96.8 ± 4.8^A^ | 45.8 ± 0.8^a^ | 11.1 ± 0.1^C^ | 1.8 ± 1.3^bc^ | 6.7 ± 0.5^CD^ | 13.6 ± 0.8^b^ | 175.7 ± 5.4^AB^ |  | 55.0 ± 1.2^bc^ | 6.3 ± 0.2^B^ | 26.1 ± 0.9^ab^ |
| SSR-7 | D6-10 |  | 94.3 ± 4.1^A^ | 46.8 ± 0.9^a^ | 10.8 ± 0.4^C^ | 1.9 ± 1.5^abc^ | 6.5 ± 0.8^D^ | 13.3 ± 0.8^b^ | 173.6 ± 4.3^AB^ |  | 54.3 ± 0.9^c^ | 6.2 ± 0.3^B^ | 26.9 ± 0.2^a^ |

^*^extracted from 500 mg of cecal content

Values with different letters are significantly different from one another (*P* < 0.05). Grey shade: period 1.

**Supplementary Table 4**. 16S rRNA gene copy numbers of specific bacterial groups in reactor effluents after initial stabilisation phase and chicken cecal content by qPCR

|  | **Day** | **Log10 16S rRNA gene copies of taxon/mL (mean ± SD)** | | | | | | |
| --- | --- | --- | --- | --- | --- | --- | --- | --- |
|  |  | **Total bacteria** | **Firmicutes** | ***Ruminococcaceae*** | ***Lactobacillus-Leuconostoc-Pediococcus spp*** | **Bacteroidetes** | ***Enterobacteriaceae*** | ***Bifidobacteriaceae*** |
| Cecal inoculum 1^*^ |  | 11.5 | 11.2 | 11.1 | 10.4 | 10.1 | 9.6 | 6.3 |
| **F1-A** | D7 – 13 | 10.4 ± 0.1^A^ | 10.2 ± 0.1^a^ | 10.0 ± 0.1^A^ | 6.3 ± 0.1^a^ | 9.9 ± 0.1^A^ | 7.5 ± 0.2^a^ | 6.8 ± 0.8^A^ |
| **F1-B** | D7 – 13 | 10.4 ± 00^A^ | 10.2 ± 0.1^a^ | 10.1 ± 0.1^A^ | 8.2 ± 0.3^b^ | 9.3 ± 0.1^B^ | 7.6 ± 0.2^a^ | 7.8 ± 0.5^B^ |
| Cecal inoculum 2^*^ |  | 11.3 | 10.8 | 10.5 | 9.5 | 9.4 | 7.4 | 6.2 |
| **F2** (IR) | D20 – 70 | 10.6 ± 0.1 | 10.0 ± 0.1 | 10.0 ± 0.2 | 6.6 ± 0.7 | 9.4 ± 0.2 | 8.0 ± 0.6 | 8.0 ± 0.7 |
| **F2** (IR) | D25-29 | 10.7 ± 0.1^A^ | 10.1 ± 0.1^a^ | 10.1 ± 0.1^A^ | 7.5 ± 0.2^ab^ | 9.5 ± 0.2^A^ | 7.9 ± 0.1^a^ | 7.1 ± 0.8^A^ |
| SSR-1 | D6-10 | 10.7 ± 0.1^A^ | 10.1 ± 0.0 ^a^ | 10.2 ± 0.0^A^ | 7.4 ± 0.2^ab^ | 9.6 ± 0.0^A^ | 6.8 ± 0.1^b^ | 6.9 ± 0.2^A^ |
| SSR-2 | D6-10 | 10.7 ± 0.0^A^ | 10.1 ± 0.1 ^a^ | 10.2 ± 0.1^A^ | 7.7 ± 0.1^a^ | 9.4 ± 0.1^A^ | 6.9 ± 0.2^b^ | 6.8 ± 0.3^A^ |
| SSR-3 | D6-10 | 10.7 ± 0.1^A^ | 10.1 ± 0.0 ^a^ | 10.2 ± 0.0^A^ | 7.2 ± 0.3^b^ | 10.1 ± 0.1^B^ | 6.8 ± 0.1^b^ | 6.6 ± 0.3^A^ |
| SSR-4 | D6-10 | 10.6 ± 0.0^A^ | 10.1 ± 0.0 ^a^ | 10.2 ± 0.0^A^ | 7.5 ± 0.3^ab^ | 8.3 ± 0.3^c^ | 6.9 ± 0.2^b^ | 7.2 ± 0.2^A^ |
| Cecal inoculum 3* |  | 11.5 | 11.0 | 11.3 | 9.9 | 7.9 | 8.3 | 6.8 |
| **F3 (IR)** | D16-82 | 10.5 ± 0.2 | 10.7 ± 0.4 | 9.8 ± 0.3 | 7.9 ± 0.5 | 5.9 ± 0.2 | 10.4 ± 0.2 | 6.3 ± 0.2 |
| **F3 (IR)** | D29-33 | 10.5 ± 0.1^A^ | 10.5 ± 0.1^b^ | 9.8 ± 0.1^B^ | 7.5 ± 0.3^a^ | 5.8 ± 0.2^CD^ | 10.6 ± 0.2^a^ | 6.2 ± 0.2^AB^ |
| SSR-1 | D6-10 | 10.5 ± 0.1^A^ | 10.5 ± 0.1^b^ | 9.9 ± 0.1^AB^ | 7.6 ± 0.4^a^ | 6.1 ± 0.2^BC^ | 10.4 ± 0.2^ab^ | 6.2 ± 0.1^AB^ |
| SSR-2 | D6-10 | 10.5 ± 0.1^A^ | 10.5 ± 0.1^b^ | 9.9 ± 0.1^AB^ | 7.5 ± 0.4^a^ | 5.6 ± 0.1^D^ | 9.3 ± 0.4^cd^ | 6.1 ± 0.1^B^ |
| SSR-3 | D6-10 | 10.5 ± 0.1^A^ | 10.5 ± 0.1^b^ | 9.9 ± 0.1^AB^ | 6.7 ± 0.3^b^ | 5.8 ± 0.2^CD^ | 8.8 ± 0.6^a^ | 6.1 ± 0.2^AB^ |
| SSR-4 | D6-10 | 10.6 ± 0.1^A^ | 10.6 ± 0.1^ab^ | 10.1 ± 0.2^AB^ | 7.2 ± 0.4^ab^ | 6.4 ± 0.1^AB^ | 10.5 ± 0.1^a^ | 6.3 ± 0.2^AB^ |
| SSR-5 | D6-10 | 10.7 ± 0.1^A^ | 10.7 ± 0.1^a^ | 10.1 ± 0.1^A^ | 5.9 ± 0.3^c^ | 6.6 ± 0.1^A^ | 10.5 ± 0.1^a^ | 6.4 ± 0.2^A^ |
| SSR-6 | D6-10 | 10.6 ± 0.1^A^ | 10.6 ± 0.1^ab^ | 10.1 ± 0.1^AB^ | 7.6 ± 0.2^a^ | 6.1 ± 0.1^AB^ | 9.7 ± 0.4^bc^ | 6.3 ± 0.1^AB^ |
| SSR-7 | D6-10 | 10.6 ± 0.2^A^ | 10.6 ± 0.1^ab^ | 9.9 ± 0.1^AB^ | 6.6 ± 0.3^b^ | 6.1 ± 0.1^AB^ | 9.1 ± 0.5^cd^ | 6.2 ± 0.2^AB^ |

**^*^**Data are mean Log10 copies 16S rRNA gene g^-1^ of cecal inoculum used for fermentation.

Values with different letters are significantly different from one another (*P < 0.05*). Gray shade: after initial stabilisation in period 1.

**Supplementary Table 5**. Summary microbial phyla and the most abundant (>1) bacterial genus obtained by V3 or V4 region 16S rRNA gene amplicon sequencing in the cecal inoculum and reactor effluent samples of different fermentation after initial stabilisation. Values < 1 in all reactors are summarised in the group “Others”.

| **Taxonomy*** | **Cecal inoculum 1** | **F 1-A**  **(Day 7-13) (%)** | **F1-B**  **(Day 7-13) (%)** | **Cecal inoculum 2** | **F2**  **(Day 20-70) (%)** | **Cecal inoculum 3** | **F3**  **(Day 16-82) (%)** |
| --- | --- | --- | --- | --- | --- | --- | --- |
| **Firmicutes** | **89.4.0 ± 0.0** | **44.0 ± 3.9^a^** | **71.4 ± 6.4^b^** | **95.9 ± 0.0** | **70.3 ± 8.2** | **98.2 ± 0.0** | **65.7 ± 7.3** |
| *c_Bacilli_RF39* | 1.8 ± 0.0 | 0.7 ± 0.2 | 1.0 ± 0.5 | 2.5 ± 0.0 | 2.4 ± 0.8 | 0.0 ± 0.0 | 0.0 ± 0.0 |
| *o_Oscillospirales* | 1.2 ± 0.0 | 0.0 ± 0.0 | 0.0 ± 0.0 | 0.7 ± 0.0 | 0.1 ± 0.1 | 1.7 ± 0.0 | 0.0 ± 0.0 |
| *f_Lachnospiraceae* | 20.9 ± 0.0 | 25.8 ± 2.8 | 34.2 ± 12.3 | 23.8 ± 0.0 | 22.9 ± 5.9 | 8.2 ± 0.0 | 5.2 ± 1.9 |
| *f_Lachnospiraceae [ASF356]* | 0.5 ± 0.0 | 2.0 ± 0.2 | 1.7 ± 0.8 | 0.3 ± 0.0 | 0.4 ± 0.1 | 0.0 ± 0.0 | 0.0 ± 0.0 |
| *f_Butyricicoccaceae* | 3.1 ± 0.0 | 0.1 ± 0.1 | 0.0 ± 0.0 | 0.6 ± 0.0 | 0.0 ± 0.0 | 0.0 ± 0.0 | 0.0 ± 0.0 |
| *f_Oscillospiraceae* | 1.67 ± 0.0 | 2.8 ± 0.9 | 1.2 ± 0.9 | 1.1 ± 0.0 | 0.5 ± 0.2 | 0.6 ± 0.0 | 0.6 ± 0.7 |
| *f_Ruminococcaceae* | 3.53 ± 0.0 | 1.0 ± 0.3 | 0.6 ± 0.2 | 1.1 ± 0.0 | 0.9 ± 0.3 | 3.3 ± 0.0 | 17.1 ± 3.0 |
| *f_Ruminococcaceae [DTU089]* | 1.8 ± 0.0 | 0.1 ± 0.0 | 0.0 ± 0.0 | 0.7 ± 0.0 | 0.1 ± 0.0 | 0.3 ± 0.0 | 0.0 ± 0.0 |
| *f_Ruminococcaceae [uncultured]* | 0.2 ± 0.0 | 1.0 ± 0.3 | 0.0 ± 0.0 | 0.2 ± 0.0 | 0.3 ± 0.2 | 0.1 ± 0.0 | 0.3 ± 0.1 |
| *f_Oscillospiraceae_UCG-005* | 0.6 ± 0.0 | 0.0 ± 0.0 | 0.0 ± 0.0 | 2.0 ± 0.0 | 0.0 ± 0.0 | 0.0 ± 0.0 | 0.0 ± 0.0 |
| *g_Bacillaceae* | 1.1 ± 0.0 | 0.0 ± 0.0 | 0.0 ± 0.0 | 5.0 ± 0.0 | 0.0 ± 0.0 | 0.0 ± 0.0 | 0.0 ± 0.0 |
| *g_Erysipelatoclostridium* | 2 ± 0.0 | 0.1 ± 0.0 | 0.1 ± 0.0 | 2.1 ± 0.0 | 0.1 ± 0.1 | 0.7 ± 0.0 | 0.7 ± 0.7 |
| *g_Enterococcus* | 0.5 ± 0.0 | 1.3 ± 0.5 | 2.5 ± 2.3 | 0.1 ± 0.0 | 12.1 ± 6.3 | 0.0 ± 0.0 | 0.0 ± 0.0 |
| *g_Lactobacillus* | 17.8 ± 0.0 | 0.0 ± 0.0 | 0.7 ± 0.3 | 10.4 ± 0.0 | 0.1 ± 0.1 | 28.4 ± 0.0 | 0.5 ± 0.4 |
| *g_Streptococcus* | 0.0 ± 0.0 | 0.0 ± 0.0 | 0.0 ± 0.0 | 0.1 ± 0.0 | 0.0 ± 0.0 | 10.5 ± 0.0 | 0.0 ± 0.0 |
| *g_Clostridia_UCG-014* | 2.3 ± 0.0 | 0.7 ± 0.3 | 0.0 ± 0.0 | 1.3 ± 0.0 | 0.3 ± 0.3 | 4.0 ± 0.0 | 1.8 ± 0.9 |
| *g_Clostridia_vadinBB60_group* | 2.1 ± 0.0 | 0.0 ± 0.0 | 0.0 ± 0.0 | 2.7 ± 0.0 | 0.0 ± 0.0 | 3.6 ± 0.0 | 0.3 ± 0.3 |
| *g_Eubacterium* | 0.0 ± 0.0 | 0.1 ± 0.0 | 0.0 ± 0.0 | 0.0 ± 0.0 | 1.9 ± 2.0 | 0.0 ± 0.0 | 0.0 ± 0.0 |
| *g_Anaerostignum* | 0.0 ± 0.0 | 0.0 ± 0.0 | 0.0 ± 0.0 | 0.0 ± 0.0 | 0.0 ± 0.0 | 0.2 ± 0.0 | 0.7 ± 1.1 |
| *g_Blautia* | 0.0 ± 0.0 | 0.0 ± 0.0 | 0.0 ± 0.0 | 0.0 ± 0.0 | 0.0 ± 0.0 | 2.6 ± 0.0 | 1.3 ± 0.7 |
| *g_Dorea* | 0.0 ± 0.0 | 0.0 ± 0.0 | 0.0 ± 0.0 | 0.0 ± 0.0 | 0.0 ± 0.0 | 1.5 ± 0.0 | 0.0 ± 0.0 |
| *g_Eisenbergiella* | 0.0 ± 0.0 | 0.0 ± 0.0 | 0.0 ± 0.0 | 0.0 ± 0.0 | 0.0 ± 0.0 | 0.0 ± 0.0 | 3.3 ± 4.8 |
| *g_Lachnoclostridium* | 0.0 ± 0.0 | 0.0 ± 0.0 | 0.0 ± 0.0 | 0.0 ± 0.0 | 0.0 ± 0.0 | 0.8 ± 0.0 | 3.5 ± 0.8 |
| *g_Lachnospira* | 0.0 ± 0.0 | 0.0 ± 0.0 | 0.0 ± 0.0 | 0.0 ± 0.0 | 0.0 ± 0.0 | 0.0 ± 0.0 | 1.5 ± 1.0 |
| *g_Sellimonas* | 0.9 ± 0.0 | 0.2 ± 0.0 | 0.5 ± 0.3 | 0.7 ± 0.0 | 0.3 ± 0.1 | 1.8 ± 0.0 | 1.2 ± 0.4 |
| *g_Tyzzerella* | 0.0 ± 0.0 | 0.0 ± 0.0 | 0.0 ± 0.0 | 0.0 ± 0.0 | 0.0 ± 0.0 | 0.0 ± 0.0 | 4.3 ± 2.0 |
| *g_[Ruminococcus]_torques_group* | 0.0 ± 0.0 | 0.0 ± 0.0 | 0.0 ± 0.0 | 0.0 ± 0.0 | 0.0 ± 0.0 | 10.2 ± 0.0 | 8.2 ± 2.8 |
| *g_Butyricicoccus* | 1.1 ± 0.0 | 0.1 ± 0.0 | 0.0 ± 0.0 | 6.4 ± 0.0 | 0.0 ± 0.0 | 3.6 ± 0.0 | 0.2 ± 0.1 |
| *g_Flavonifractor* | 0.3 ± 0.0 | 0.3 ± 0.2 | 0.1 ± 0.1 | 0.1 ± 0.0 | 0.6 ± 1.2 | 0.2 ± 0.0 | 1.2 ± 0.8 |
| *g_Intestinimonas* | 0.7 ± 0.0 | 0.9 ± 0.2 | 2.0 ± 0.9 | 1.4 ± 0.0 | 1.0 ± 0.6 | 0.3 ± 0.0 | 0.0 ± 0.0 |
| *g_Oscillospira* | 0.2 ± 0.0 | 0.4 ± 0.1 | 0.0 ± 0.0 | 0.1 ± 0.0 | 1.1 ± 0.4 | 0.0 ± 0.0 | 0.1 ± 0.1 |
| *g_Pseudoflavonifractor* | 0.1 ± 0.0 | 0.1 ± 0.0 | 1.0 ± 0.5 | 0.0 ± 0.0 | 1.3 ± 0.7 | 0.0 ± 0.0 | 0.2 ± 0.2 |
| *g_Faecalibacterium* | 17.1 ± 0.0 | 0.0 ± 0.0 | 0.0 ± 0.0 | 19.9 ± 0.0 | 0.0 ± 0.0 | 5.9 ± 0.0 | 0.0 ± 0.0 |
| *g_Fournierella* | 1.0 ± 0.0 | 0.0 ± 0.0 | 22.4 ± 17.1 | 1.3 ± 0.0 | 17.5 ± 7.1 | 0.0 ± 0.0 | 0.5 ± 0.3 |
| *g_Incertae_Sedis* | 0.8 ± 0.0 | 0.0 ± 0.0 | 0.1 ± 0.1 | 1.1 ± 0.0 | 1.3 ± 0.6 | 0.5 ± 0.0 | 2.7 ± 1.0 |
| *g_Negativibacillus* | 0.4 ± 0.0 | 0.8 ± 0.2 | 1.1 ± 0.6 | 0.2 ± 0.0 | 0.8 ± 0.3 | 0.3 ± 0.0 | 1.5 ± 1.0 |
| *g_Subdoligranulum* | 0.6 ± 0.0 | 0.1 ± 0.1 | 0.4 ± 0.3 | 1.6 ± 0.0 | 0.2 ± 0.4 | 1.5 ± 0.0 | 0.0 ± 0.1 |
| *g_Romboutsia* | 0.0 ± 0.0 | 0.0 ± 0.0 | 0.0 ± 0.0 | 0.0 ± 0.0 | 0.0 ± 0.0 | 1.8 ± 0.0 | 0.0 ± 0.0 |
| **Bacteroidetes** | **4.6 ± 0.0** | **34.9 ± 5.2^a^** | **6.3 ± 2.9^b^** | **3.5 ± 0.0** | **9.1 ± 4.2** | **0.2 ± 0.0** | **0.0 ± 0.0** |
| *g_Bacteroides* | 4.2 ± 0.0 | 34.9 ± 5.2 | 6.3 ± 2.9 | 1.4 ± 0.0 | 9.1 ± 4.2 | 0.0 ± 0.0 | 0.0 ± 0.0 |
| *g_Alistipes* | 0.4 ± 0.0 | 0.0 ± 0.0 | 0 ± 0 | 2.1 ± 0.0 | 0.0 ± 0.0 | 0.2 ± 0.0 | 0.0 ± 0.0 |
| **Proteobacteria** | **5.4 ± 0.0** | **20.8 ± 7.2^a^** | **21.9 ± 7.2^b^** | **0.3 ± 0.0** | **17.6 ± 9.5** | **0.3 ± 0.0** | **33.6 ± 7.2** |
| *g_Escherichia-Shigella* | 5.4 ± 0.0 | 0.5 ± 0.2 | 0.6 ± 0.1 | 0.1 ± 0.0 | 3.1 ± 3.4 | 0.0 ± 0.0 | 12.2 ± 4.5 |
| *g_Proteus* | 0.0 ± 0.0 | 20.3 ± 7.3 | 20.1 ± 6.5 | 0.1 ± 0.0 | 14.5 ± 10.2 | 0.1 ± 0.0 | 21.3 ± 8 |
| *g_Pseudomonas* | 0.0 ± 0.0 | 0.0 ± 0.0 | 1.2 ± 1.6 | 0 ± 0.0 | 0.0 ± 0.0 | 0.1 ± 0.0 | 0.0 ± 0.0 |
| **Actinobacteria** | **0.1 ± 0.0** | **0.2 ± 0.1^a^** | **0.5 ± 0.3^b^** | **0.3 ± 0.0** | **2.9± 2.8** | **0.7 ± 0.0** | **0.6 ± 0.3** |
| *g_Bifidobacterium* | 0.0 ± 0.0 | 0.1 ± 0.1 | 0.4 ± 0.3 | 0 ± 0.0 | 2.8 ± 2.9 | 0.0 ± 0.0 | 0.0 ± 0.0 |
| **Others** | **5.8 ± 0.0** | 3.5 ± 1.5 | **0.7 ± 0.3** | **7.9 ± 0.0** | **1.8 ± 0.5** | **6 ± 0.0** | **4.8 ± 1.1** |

*When assignment at the genus level was not possible, the highest-level taxonomy was presented. Values with different letters are significantly different from one another (*P* < 0.01).

**Supplementary Table 6**. Summary microbial phyla and the most abundant (>1) bacterial genus obtained by V3 region 16S rRNA gene amplicon sequencing of samples from inoculum reactor (IR) and second-stage reactors (SSRs) of F2 after initial stabilisation in period 1. Values < 1 in all reactors are summarised in the group “Others”.

| **Taxonomy*** | **F2 (IR)**  **(Day 25-29) (%)** | **SSR-1**  **(Day 6-10) (%)** | **SSR-2**  **(Day 6-10) (%)** | **SSR-3**  **(Day 6-10) (%)** | **SSR-4**  **(Day 6-10) (%)** |
| --- | --- | --- | --- | --- | --- |
| **Firmicutes** | **60.7 ± 0.1^cd^** | **69.8 ± 0.0^ab^** | **76.7 ± 0.0^b^** | **53.1 ± 0.0^d^** | **89.6 ± 0.0^a^** |
| *c_Bacilli [RF39]* | 0.2 ± 0.2^B^ | 0.3 ± 0.1^B^ | 0.5 ± 0.3^AB^ | 0.1 ± 0.0^B^ | 1.0 ± 0.6^A^ |
| *o_Oscillospirales* | 1.5 ± 0.9^b^ | 2.8 ± 0.6^a^ | 3.0 ± 0.4^a^ | 2.6 ± 0.3^a^ | 2.3 ± 0.4^ab^ |
| *f_Lachnospiraceae* | 20.8 ± 6.3^B^ | 27.3 ± 1.7^AB^ | 30 ± 2.2^A^ | 21.4 ± 1.6^B^ | 29.5 ± 3.3^A^ |
| *f_Lachnospiraceae [ASF356]* | 0.4 ± 0.1^b^ | 0.3 ± 0.1^b^ | 0.4 ± 0.1^ab^ | 0.2 ± 0.0^b^ | 0.7 ± 0.3^a^ |
| *f_Ruminococcaceae* | 0.5 ± 0.2^C^ | 1.6 ± 0.2^A^ | 1.6 ± 0.2^AB^ | 1.3 ± 0.1^B^ | 1.8 ± 0.2^A^ |
| *f_Ruminococcaceae [uncultured]* | 0.3 ± 0.3^b^ | 0.8 ± 0.2^ab^ | 0.8 ± 0.2^a^ | 0.7 ± 0.1^ab^ | 0.7 ± 0.4^ab^ |
| *g_Clostridia_UCG-014* | 0.5 ± 0.2^B^ | 0.8 ± 0.4^AB^ | 0.9 ± 0.3^AB^ | 0.5 ± 0.3B | 1.2 ± 0.4^A^ |
| *g_Anaerostignum* | 2.2 ± 0.8^a^ | 2.9 ± 0.9^a^ | 2.6 ± 1.2^a^ | 3.5 ± 0.2^a^ | 1.8 ± 1.1^a^ |
| *g_Enterococcus* | 13.6 ± 6.9^A^ | 2.2 ± 0.3^B^ | 3.4 ± 0.9^B^ | 2.0 ± 0.4^B^ | 4.5 ± 1.4^B^ |
| *g_Acetanaerobacterium* | 0.2 ± 0.1^b^ | 1.1 ± 0.2^a^ | 1.3 ± 0.4^a^ | 0.4 ± 0.1^b^ | 0.4 ± 0.2^b^ |
| *g_Flavonifractor* | 0.2 ± 0.1^A^ | 1.3 ± 0.8^A^ | 0.8 ± 0.6^A^ | 1.2 ± 1.0^A^ | 0.3 ± 0.1^A^ |
| *g_Intestinimonas* | 1.1 ± 1.0^b^ | 3.2 ± 0.4^a^ | 3.6 ± 0.2^a^ | 1.5 ± 0.1^b^ | 4.0 ± 0.6^a^ |
| *g_Oscillospira* | 0.9 ± 0.5^B^ | 1.1 ± 0.3^AB^ | 1.3 ± 0.4^A^ | 0.4 ± 0.1^B^ | 1.3 ± 0.6^A^ |
| *g_Pseudoflavonifractor* | 1.2 ± 1.0^d^ | 3.2 ± 0.3^bc^ | 3.6 ± 0.3^ab^ | 2.4 ± 0.1^c^ | 4.3 ± 0.4^a^ |
| *g_Fournierella* | 13.2 ± 3.6^BC^ | 15.7 ± 2.3^BC^ | 16.3 ± 3.2^B^ | 10.7 ± 2.2^C^ | 28.4 ± 1.9^A^ |
| *g_Incertae_Sedis* | 0.9 ± 0.1^c^ | 1.7 ± 0.2^b^ | 1.9 ± 0.1^b^ | 1.1 ± 0.1^c^ | 2.8 ± 0.3^a^ |
| **Bacteroidetes** | **6.5 ± 0.0^CD^** | **15.6 ± 0.0^B^** | **10.5 ± 0.0^AB^** | **40 ± 0.0^A^** | **1.1 ± 0.0^D^** |
| *g_Bacteroides* | 6.5 ± 2.8^c^ | 15.5 ± 1.5^b^ | 10.5 ± 2.4^bc^ | 40 ± 4.9^a^ | 1.1 ± 0.6^d^ |
| **Proteobacteria** | **31.9 ± 0.1^A^** | **13 ± 0.0^B^** | **12.5 ± 0.0^B^** | **6.7 ± 0.0^B^** | **8.8 ± 0.0^B^** |
| *g_Escherichia-Shigella* | 1.1 ± 0.5^a^ | 0.1 ± 0.1^b^ | 0.1 ± 0.0^b^ | 0.1 ± 0.0^b^ | 0.2 ± 0.1^b^ |
| *g_Proteus* | 30.8 ± 13.9^A^ | 12.9 ± 2.3^B^ | 12.4 ± 3.4^B^ | 6.6 ± 2.8^B^ | 8.6 ± 1.7^B^ |
| **Actinobacteria** | **1 ± 0.0^a^** | **1.6 ± 0.0^a^** | **0.3 ± 0.0^a^** | **0.1 ± 0.0^a^** | **0.4 ± 0.0^a^** |
| *c_Actinobacteria* | 0.0 ± 0.0^A^ | 1.4 ± 1.9^A^ | 0.0 ± 0.0^A^ | 0.0 ± 0.0^A^ | 0.0 ± 0.0^A^ |
| *g_Bifidobacterium* | 0.8 ± 1.4**^a^** | 0.1 ± 0.1**^a^** | 0.1 ± 0.1**^a^** | 0.0 ± 0.0**^a^** | 0.3 ± 0.1**^a^** |
| **Others** | **3.3 ± 0.7^B^** | **3.7 ± 0.3^B^** | **4.8 ± 0.3^A^** | **3.2 ± 0.3^B^** | **4.8 ± 0.3^A^** |

*When assignment at the genus level was not possible, the highest-level taxonomy was presented.

Values with different letters are significantly different from one another (P < 0.05)

**Supplementary Table 7**. Summary microbial phyla and the most abundant (>1) bacterial genus obtained by V4 region 16S rRNA gene amplicon sequencing of samples from inoculum reactor (IR) and second-stage reactors (SSRs) of F3 after initial stabilisation in period 1. Values < 1 in all reactors are summarised in the group “Others”.

| **Taxonomy*** | **F3 (IR)**  **(Day 29-33) (%)** | **SSR-1**  **(Day 6-10) (%)** | **SSR-2**  **(Day 6-10) (%)** | **SSR-3**  **(Day 6-10) (%)** | **SSR-4**  **(Day 6-10) (%)** | **SSR-5**  **(Day 6-10) (%)** | **SSR-6**  **(Day 6-10) (%)** | **SSR-7**  **(Day 6-10) (%)** |
| --- | --- | --- | --- | --- | --- | --- | --- | --- |
| **Firmicutes** | **65.7 ± 4.2^BC^** | **74.5 ± 6.2^ABC^** | **82.7 ± 11.9^AB^** | **87.6 ± 9.2^A^** | **61.3 ± 13.7^C^** | **65.1 ± 8.8^BC^** | **76.6 ± 7.4^ABC^** | **78.0 ± 9.3^ABC^** |
| *g_Bacillus* | 0.0 ± 0.0^b^ | 0.0 ± 0.0^b^ | 13.1 ± 2.7^a^ | 15.4 ± 3.9^a^ | 0.8 ± 0.9^b^ | 0.7 ± 0.5^b^ | 0.0 ± 0.0^b^ | 0.0 ± 0.0^b^ |
| *g_Erysipelatoclostridium* | 2.4 ± 0.4^A^ | 3.3 ± 1.3^A^ | 2.1 ± 0.5^A^ | 1.9 ± 0.4^A^ | 2.2 ± 1.4^A^ | 2.1 ± 0.4^A^ | 2.8 ± 0.9^A^ | 2.1 ± 0.2^A^ |
| *g_Enterococcus* | 0.0 ± 0.1^a^ | 1.1 ± 0.5^a^ | 0.5 ± 0.1^a^ | 0.0 ± 0.0^a^ | 3.3 ± 5.9^a^ | 0.2 ± 0.1^a^ | 0.0 ± 0.0^a^ | 0.0 ± 0.0^a^ |
| *g_Clostridia [UCG-014]* | 1.5 ± 0.3^C^ | 2.0 ± 0.3^BC^ | 2.5 ± 0.8^BC^ | 2.5 ± 0.4^BC^ | 1.6 ± 1.4^C^ | 1.2 ± 0.2^C^ | 3.4 ± 1.2^AB^ | 4.8 ± 0.9^A^ |
| *g_Defluviitaleaceae [UCG-011]* | 0.6 ± 0.2^ab^ | 1.0 ± 0.2^a^ | 0.1 ± 0.2^b^ | 0.6 ± 0.1^ab^ | 0.9 ± 0.8^a^ | 0.6 ± 0.2^ab^ | 0.4 ± 0.2^ab^ | 0.6 ± 0.1^ab^ |
| *f_Lachnospiraceae* | 8.0 ± 1.6^A^ | 7.4 ± 1.1^A^ | 5.1 ± 1.8^A^ | 6.2 ± 0.9^A^ | 5.7 ± 3.2^A^ | 5.8 ± 0.3^A^ | 7.3 ± 0.9^A^ | 5.4 ± 0.3^A^ |
| *g_Anaerostipes* | 0.2 ± 0.2^b^ | 0.2 ± 0.2^b^ | 0.2 ± 0.1^b^ | 0.8 ± 0.4^a^ | 0.2 ± 0.3^b^ | 0.3 ± 0.2^b^ | 0.4 ± 0.3^ab^ | 0.5 ± 0.4^ab^ |
| *g_Blautia* | 1.9 ± 0.1^A^ | 2.4 ± 0.4^A^ | 2.4 ± 0.6^A^ | 2.7 ± 0.5^A^ | 1.7 ± 1.0^A^ | 1.8 ± 0.4^A^ | 2.1 ± 0.5^A^ | 1.8 ± 0.6^A^ |
| *g_Eisenbergiella* | 0.8 ± 0.3^b^ | 1.6 ± 0.6^b^ | 1.7 ± 0.8^b^ | 2.0 ± 0.8^b^ | 1.4 ± 0.9^b^ | 1.7 ± 0.5^b^ | 6.5 ± 1.2^a^ | 5.4 ± 0.6^a^ |
| *g_Lachnoclostridium* | 4.0 ± 0.8^A^ | 3.8 ± 0.7^A^ | 3.8 ± 0.7^A^ | 3.2 ± 0.3^A^ | 2.9 ± 1.8^A^ | 3.5 ± 0.6^A^ | 3.4 ± 0.4^A^ | 2.7 ± 0.4^A^ |
| *g_Lachnospira* | 1.6 ± 0.8^c^ | 2.9 ± 0.8^abc^ | 3.3 ± 0.9^ab^ | 4.1 ± 0.2^a^ | 2.4 ± 1.4^bc^ | 2.9 ± 0.5^abc^ | 3.0 ± 0.1^abc^ | 3.8 ± 0.8^ab^ |
| *g_Sellimonas* | 1.0 ± 0.4^B^ | 1.2 ± 0.2^AB^ | 1.7 ± 0.2^A^ | 1.5 ± 0.3^AB^ | 0.9 ± 0.5^B^ | 1.5 ± 0.2^AB^ | 1.0 ± 0.2^B^ | 1.3 ± 0.2^AB^ |
| *g_Tyzzerella* | 5.5 ± 0.9^a^ | 5.2 ± 1.8^a^ | 5.0 ± 1.6^a^ | 6.0 ± 1.5^a^ | 5.8 ± 3.1^a^ | 5.1 ± 1.1^a^ | 5.3 ± 1.2^a^ | 6.5 ± 1.2^a^ |
| *g_[Eubacterium]_hallii_group* | 1.0 ± 0.2^ABC^ | 1.2 ± 0.4^AB^ | 1.3 ± 0.2^A^ | 1.3 ± 0.4^AB^ | 0.5 ± 0.4^C^ | 0.6 ± 0.1^C^ | 0.8 ± 0.1B^C^ | 0.7 ± 0.1^C^ |
| *g_[Ruminococcus]_torques_group* | 6.9 ± 1.6^ab^ | 6.5 ± 1.3^ab^ | 7.1 ± 1.2^ab^ | 7.7 ± 2.0^ab^ | 4.1 ± 2.9^b^ | 4.8 ± 1.1^b^ | 9.5 ± 2.3^a^ | 7.5 ± 2.1^ab^ |
| *g_Colidextribacter* | 1.1 ± 0.3^BC^ | 1.4 ± 0.2^ABC^ | 1.7 ± 0.6^AB^ | 2.1 ± 0.5^A^ | 0.8 ± 0.5^C^ | 1.4 ± 0.2^ABC^ | 1.4 ± 0.3^ABC^ | 1.8 ± 0.2^AB^ |
| *g_Flavonifractor* | 1.0 ± 0.7^b^ | 1.1 ± 0.3^b^ | 3.2 ± 0.9^a^ | 2.7 ± 0.3^a^ | 0.8 ± 0.4^b^ | 1.0 ± 0.3^b^ | 3.1 ± 0.7^a^ | 3.3 ± 0.7^a^ |
| *g_Oscillibacter* | 1.7 ± 0.4^AB^ | 1.4 ± 0.2^B^ | 1.7 ± 0.5^AB^ | 2.4 ± 0.7^A^ | 1.0 ± 0.6^B^ | 1.3 ± 0.2^B^ | 1.3 ± 0.2^B^ | 1.5 ± 0.2^B^ |
| *g_Pseudoflavonifractor* | 0.1 ± 0.1^a^ | 0.9 ± 0.4^a^ | 0.7 ± 0.9^a^ | 1.0 ± 0.9^a^ | 1.1 ± 0.7^a^ | 0.9 ± 0.4^a^ | 0.7 ± 0.5^a^ | 1.0 ± 0.6 ^a^ |
| *f_Oscillospiraceae [uncultured]* | 1.0 ± 1.0^A^ | 1.7 ± 0.8^A^ | 1.9 ± 0.2^A^ | 1.5 ± 0.2^A^ | 1.4 ± 0.9^A^ | 1.9 ± 0.4^A^ | 1.3 ± 0.5^A^ | 1.6 ± 0.3^A^ |
| *f_Ruminococcaceae* | 1.5 ± 0.7^c^ | 4.7 ± 1.2^ab^ | 2.6 ± 1.1^bc^ | 2.3 ± 1.1^bc^ | 3.1 ± 3.0^abc^ | 4.1 ± 1.6^abc^ | 5.7 ± 0.9^a^ | 5.3 ± 0.7^ab^ |
| *g_Incertae_Sedis* | 3.2 ± 0.6^A^ | 2.4 ± 0.6^AB^ | 1.6 ± 0.4^B^ | 1.9 ± 0.3^B^ | 1.5 ± 0.8^B^ | 2.4 ± 0.8^AB^ | 1.7 ± 0.5^B^ | 2.0 ± 0.4^B^ |
| *g_Negativibacillus* | 0.5 ± 0.5^b^ | 0.8 ± 0.2^ab^ | 0.9 ± 0.1^ab^ | 1.0 ± 0.2^ab^ | 0.7 ± 0.5^ab^ | 1.2 ± 0.3^a^ | 0.9 ± 0.2^ab^ | 0.9 ± 0.2^ab^ |
| *g_Subdoligranulum* | 17.1 ± 3.0^A^ | 17.2 ± 4.1^A^ | 15.7 ± 3.2^A^ | 13.9 ± 3.1^A^ | 13.9 ± 3.1^A^ | 14.1 ± 1.4^A^ | 12 ± 4.5^A^ | 15.5 ± 3.4^A^ |
| **Proteobacteria** | **33.7 ± 4.2^ab^** | **24.9 ± 6.2^abc^** | **17.2 ± 12.1^bc^** | **12.2 ± 9.3^c^** | **38.5 ± 13.8^a^** | **34.7 ± 8.9^bc^** | **22.7 ± 7.4^abc^** | **21.4 ± 9.2^abc^** |
| *g_Escherichia-Shigella* | 18.1 ± 3.7^A^ | 14.9 ± 3.9^A^ | 1.8 ± 1.6^B^ | 0.8 ± 0.9^B^ | 16 ± 7.6^A^ | 12.4 ± 1.3^A^ | 3.4 ± 1.6^B^ | 1.2 ± 0.8^B^ |
| *g_Proteus* | 15.6 ± 2.7^a^ | 9.9 ± 6.7^a^ | 15.4 ± 12.7^a^ | 11.4 ± 8.4^a^ | 22.5 ± 7.8^a^ | 22.3 ± 9.9^a^ | 18.4 ± 6.9^a^ | 18.8 ± 8.7^a^ |
| **Actinobacteria** | **0.5 ± 0.4^AB^** | **0.6 ± 0.3^AB^** | **0.1 ± 0.1^B^** | **0.1 ± 0.1^B^** | **0.2 ± 0.1^B^** | **0.2 ± 0.1^AB^** | **0.9 ± 0.2^A^** | **0.6 ± 0.1^A^** |
| *g_Acinetobacter* | 0.0 ± 0.0^b^ | 0.0 ± 0.0^b^ | 0.0 ± 0.0^b^ | 0.0 ± 0.0^b^ | 0.0 ± 0.0^b^ | 0.0 ± 0.0^b^ | 0.9 ± 1.0^a^ | 1.3 ± 0.9^a^ |
| **Others** | **3.6 ± 1.2**^A^ | **3.7 ± 0.6**^A^ | **3.1 ± 0.3**^A^ | **2.9 ± 0.5**^A^ | **2.8 ± 1.5**^A^ | **4.3 ± 0.5**^A^ | **3.2 ± 0.5**^A^ | **2.9 ± 0.8**^A^ |

*When assignment at the genus level was not possible, the highest-level taxonomy was presented. Values with different letters are significantly different from one another (P < 0.05)

**References**

Bartosch, S., Fite,A., Macfarlane, G. T., & Mcmurdo, M. E. T. (2004). Characterization of Bacterial Communities in Feces from Healthy Elderly VolunteersAnd Hospitalized Elderly Patients by Using Real-Time PCRAnd Effects ofAntibiotic Treatment on the Fecal Microbiota Characterization of Bacterial Communities in Feces from.A*ppliedAnd Environmental Microbiology*, *70*(6), 3575–3581. https://doi.org/10.1128/AEM.70.6.3575

Furet, J.-P., Firmesse, O., Gourmelon, M., Bridonneau, C., Tap, J., Mondot, S., … Corthier, G. (2009). ComparativeAssessment of humanAnd farmAnimal faecal microbiota using real-time quantitative PCR. *FEMS Microbiology Ecology*, *68*(3), 351–362. https://doi.org/10.1111/j.1574-6941.2009.00671.x

Garcia-Mazcorro, J. F., Suchodolski, J. S., Jones, K. R., Clark-Price, S. C., Dowd, S. E., Minamoto, Y., … Dossin, O. (2012). Effect of the proton pump inhibitor omeprazole on the gastrointestinal bacterial microbiota of healthy dogs. *FEMS Microbiology Ecology*, *80*(3), 624–636. https://doi.org/10.1111/j.1574-6941.2012.01331.x

Guo, X., Xia, X., Tang, R., Zhou, J., Zhao, H., & Wang, K. (2008). Development ofA real-time PCR method for FirmicutesAnd Bacteroidetes in faecesAnd itsApplication to quantify intestinal population of obeseAnd lean pigs. *Letters inApplied Microbiology*, *47*(5), 367–373. https://doi.org/10.1111/j.1472-765X.2008.02408.x

Meimandipour,A., Shuhaimi, M., Soleimani,A. F.,Azhar, K., Hair-Bejo, M., Kabeir, B. M., … Yazid,A. M. (2010). Selected microbial groupsAnd short-chain fattyAcids profile inA simulated chicken cecum supplemented with two strains of Lactobacillus. *Poultry Science*, *89*(3), 470–476. https://doi.org/10.3382/ps.2009-00495

Ramirez-Farias, C., Slezak, K., Fuller, Z., Duncan,A., Holtrop, G., & Louis, P. (2009). Effect of inulin on the human gut microbiota: stimulation of BifidobacteriumAdolescentisAnd Faecalibacterium prausnitzii. *British Journal of Nutrition*, *101*(4), 533–540. https://doi.org/10.1017/S0007114508019880

Walter, J., Britton, R.A., & Roos, S. (2011). Host-microbial symbiosis in the vertebrate gastrointestinal tractAnd the Lactobacillus reuteri paradigm. *Proc NatlAcad Sci U SA*, *108 Suppl*, 4645–4652. https://doi.org/10.1073/pnas.1000099107
